# A panoramic analysis of keloid pathogenesis: multidimensional network regulation of genetics-immunity-metabolism-mechanical force

**DOI:** 10.3389/fimmu.2026.1936809

**Published:** 2026-08-26

**Authors:** Mengguo Liu

**Affiliations:** Department of Dermatology, Huashan Hospital, Fudan University, Shanghai, China

**Keywords:** genetic Susceptibility, immune microenvironment, keloid, mechanical force transduction, metabolic reprogramming

## Abstract

Keloid, as a unique form of pathological scar, involves complex interactions across multiple dimensions in its pathogenesis, including genetic susceptibility, epigenetic regulation, immune microenvironment disorders, metabolic reprogramming, abnormal mechanical force transduction, and fine-tuning by non-coding RNAs. At the genetic level, alleles such as HLA-DRB1*15, HOX gene family, and high-frequency mutation genes including MUC4 constitute the foundation of congenital susceptibility; at the epigenetic level, reduced DNA methylation and imbalanced m6A modification promote fibrosis by regulating target genes such as COL1A1. In core signaling pathways, TGF-β/Smad, MAPK/ERK, PI3K/AKT/mTOR, and Wnt/β-catenin pathways form a cross-talk network, driving persistent activation of fibroblasts and excessive extracellular matrix deposition. Characteristics of the immune microenvironment include M2-like macrophage polarization, Th17/IL-17 axis activation, and chronic low-grade inflammation maintained by inflammatory factor networks such as CXCL12. Regarding metabolic reprogramming, keloid cells exhibit “Warburg effect” features, prioritizing aerobic glycolysis while suppressing oxidative phosphorylation, alongside ferroptosis resistance and abnormal sphingolipid metabolism. Mechanical forces trigger the opening of mechanosensitive PIEZO1 ion channels, inducing abnormal calcium influx and subsequent YAP/TAZ nuclear translocation, which creates a self-amplifying vicious cycle alongside elevated matrix stiffness. Furthermore, lncRNAs, circRNAs, and miRNAs finely regulate the fibrotic process through competing endogenous RNA (ceRNA) networks and m6A modification-dependent mechanisms. The integration of these multidimensional mechanisms provides a theoretical basis for developing multi-target combination therapeutic strategies. Future research should promote the application of precision medicine in keloid management through multi-omics integration and artificial intelligence algorithms. Microenvironment; Metabolic reprogramming; Mechanical force transduction.

## 1 Introduction: panoramic integrative analysis—from single mechanism to multidimensional network

**Figure 1:**
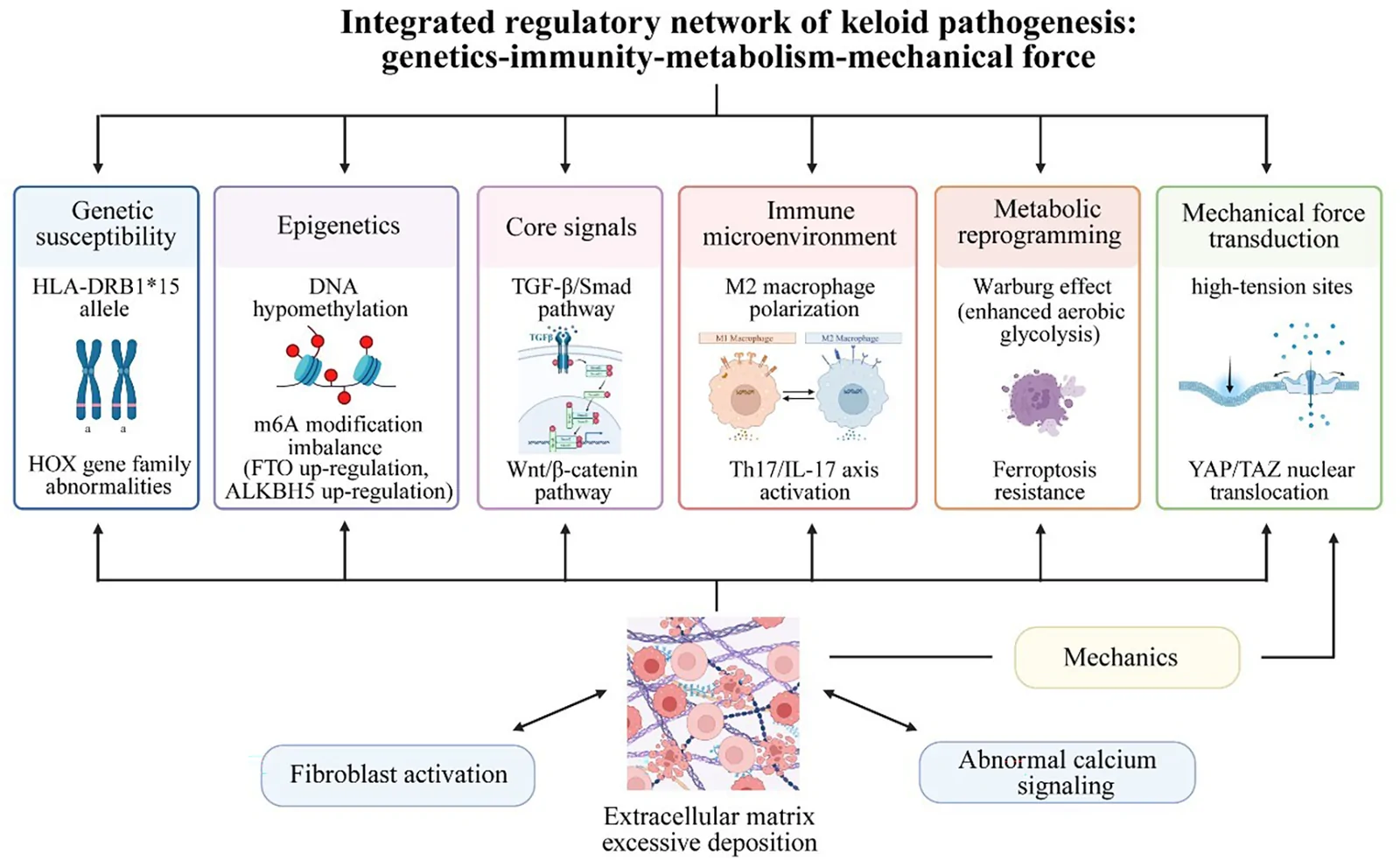
Panoramic multidimensional regulatory network of keloid pathogenesis: The occurrence and development of keloid result from the interweaving and dynamic evolution of multi-level factors including genetic background, epigenetic modifications, immune microenvironment, cellular metabolic status, mechanical force stimulation, and non-coding RNA regulation. (1) Genetic susceptibility: HLADRB1*15 alleles and HOX gene family abnormalities constitute the congenital foundation; (2) Epigenetics: DNA hypomethylation and m6A modification imbalance (such as upregulation of FTO and ALKBH5) lead to derepression of fibrotic genes; (3) Core signaling: TGF-β/Smad, Wnt/β-catenin and other pathways form cross-talk networks; (4) Immune microenvironment: M2 macrophage polarization and Th17/IL17 axis activation maintain chronic inflammation; (5) Metabolic reprogramming: Manifested as the “Warburg effect” (enhanced aerobic glycolysis) and ferroptosis resistance; (6) Mechanical force transduction: High-tension sites convert physical signals into biochemical signals through YAP/TAZ nuclear translocation and abnormal calcium signaling. These dimensions collectively drive persistent fibroblast activation and excessive extracellular matrix deposition.

Keloid is a pathological scar characterized by abnormal fibroblast proliferation and excessive extracellular matrix deposition. Its clinical manifestations feature unique invasive growth properties, extending beyond original wound boundaries to invade surrounding normal skin, with extremely high postoperative recurrence rates, causing severe physical and psychological burdens to patients. Despite significant clinical hazards, the pathogenesis of keloid has long lacked systematic explanation. Traditional research mostly focused on single signaling pathways or cytokines, struggling to explain the complex heterogeneity and refractory nature of this disease. In recent years, with rapid advances in genomics, epigenetics, single-cell sequencing technology, metabolomics, and biomechanics research, keloid pathogenesis studies are undergoing a paradigm shift from “single dimension” to “multidimensional network”. Increasing evidence indicates that keloid development results from the interweaving and dynamic evolution of multi-level factors including genetic background, epigenetic modifications, immune microenvironment, cellular metabolic status, mechanical force stimulation, and non-coding RNA regulation. This multidimensional regulatory network not only determines keloid initiation and progression but also explains its significant heterogeneity across different ethnicities, anatomical locations, and individuals. Therefore, panoramic integrative analysis of keloid pathogenesis and constructing multidimensional regulatory networks from molecular, cellular, tissue to system levels hold important theoretical value and clinical significance for breaking current therapeutic bottlenecks and developing precise intervention strategies. This review aims to systematically summarize the latest advances in keloid research, break through traditional single-mechanism limitations, and for the first time construct an integrated regulatory network for keloid pathogenesis from genetic-immune-metabolicmechanical force multidimensional perspectives (**Figure 1**), providing new theoretical frameworks for mechanism analysis and clinical translation of this refractory disease.

## 2 Genetic susceptibility: the congenital foundation of keloid pathogenesis

### 2.1 Ethnic differences and genetic polymorphism

Keloid demonstrates significant ethnic differences, with incidence rates notably higher in African, Asian, and Hispanic populations compared to Caucasian populations. This epidemiological feature strongly suggests the decisive role of genetic factors in disease occurrence (1). Genome-wide association studies (GWAS) and candidate gene analyses have identified multiple genetic loci associated with keloid susceptibility, involving gene families such as the human leukocyte antigen (HLA) system, vitamin D receptor (VDR), and neural precursor cell expressed developmentally down-regulated gene 4-like protein (NEDD4) signaling cascade (1). Among these, the HLA-DRB1*15 allele has been confirmed to associate with increased keloid risk across three different racial groups, while some HLA class I alleles show population-specific association patterns, reflecting the impact of genetic heterogeneity on disease phenotypes (1). The first whole-exome sequencing study in the Chinese population revealed specific mutation spectrum characteristics of keloid: 3,125 single nucleotide variations (SNVs) and 299 insertion-deletion mutations (InDels) were identified in 9 patients, with high frequency mutation genes including mucin 4 (MUC4, 55.6%), tubulin tyrosine ligaselike protein 12 (TTLL12, 33.3%), calcium voltage-gated channel subunit alpha1 C (CACNA1C, 33.3%), and mucin 12 (MUC12, 33.3%), with a mean tumor mutation burden (TMB) as high as 289 mutations per megabase pair (2). These mutations were mainly enriched in cancer-related microRNAs and calcium signaling pathways, providing new perspectives for understanding the molecular characteristics of keloid in the Chinese population (2).

### 2.2 Homeobox genes and positional specificity

The role of homeobox (HOX) gene family in keloid pathogenesis has received increasing attention. Early studies reported reduced expression of multiple HOX genes in keloid fibroblasts, but this finding may have been confounded by sampling site differences (3). When strictly matching sampling sites between keloid and normal skin, expression differences in HOXA7, HOXA9, and HOXC8 were not significant, suggesting that previously observed HOX gene downregulation may reflect anteriorposterior axis positional identity differences rather than disease-specific changes (3). However, the functional relevance of HOX genes cannot be ignored: HOXA9 overexpression, though not affecting fibroblast proliferation, significantly inhibited cell migration and altered gene expression profiles, suggesting that HOX genes may play regulatory roles in keloid predilection sites (ears, face, upper trunk) (3). Based on weighted gene co-expression network analysis (WGCNA) and LASSO algorithms, HOXD3 and UNC5C were identified as characteristic molecular markers of keloid, with UNC5C correlating with various immune cell infiltration levels, while HOXD3 participates in regulating key biological processes such as neurite outgrowth, oncogenic MAPK signaling, and chemical synaptic transmission (4).

### 2.3 Chromosomal variations and genomic instability

Keloid cells exhibit tumor-like genomic instability characteristics. Genome-wide analysis revealed extensive copy number variations (CNVs) in keloid tissue, with a total of 67,873 CNVs identified, involving multiple gene regions related to cell proliferation, differentiation, and matrix metabolism (2). Notably, telomerase activity is abnormally elevated in keloid fibroblasts: PAX5 activates telomerase activity by transcriptionally regulating SND1 (staphylococcal nuclease domain-containing protein 1), promoting keloid formation in burn-injured skin (5). SND1 is significantly upregulated in both keloid tissue and fibroblasts; its overexpression enhances cell proliferation, colony formation ability, telomerase activity, pro-inflammatory factor production, and fibrotic gene expression, effects mediated through the ERK/JNK signaling pathway (5). This finding explains the molecular basis of the immortalized-like proliferation characteristics of keloid cells and provides a basis for developing telomerase-targeted therapeutic strategies.

Genetic variants alone are insufficient to directly trigger keloid lesions; they serve as static congenital predispositions that require dynamic epigenetic reprogramming to unlock pathological gene expression programs, forming an indispensable upstreamdownstream regulatory axis linking genetic background to downstream fibrotic phenotypes. The genomic instability induced by widespread CNVs and high tumor mutation burden described above impairs the fidelity of DNA modification and chromatin remodeling machinery, making keloid-prone fibroblasts more susceptible to aberrant methylation and RNA modification upon skin injury. Conversely, epigenetic modifications act as reversible molecular switches that translate fixed genetic sequences into context-dependent transcriptional responses. This genetic-epigenetic crosstalk bridges the static germline susceptibility and dynamic post-injury molecular changes, and further couples with immune, metabolic and mechanical signaling cascades elaborated in subsequent sections to drive full keloid progression.

## 3 Epigenetic regulation: the layer of genetic information beyond DNA sequence

### 3.1 DNA methylation and chromatin remodeling

Epigenetic modifications play a central regulatory role in keloid pathogenesis. Genome-wide DNA methylation analysis revealed significantly lower methylation levels of long interspersed nuclear element-1 (LINE-1) and Alu repetitive sequences in keloid tissue compared to normal skin (P<0.0001 and P = 0.0147), with this hypomethylation state negatively correlated with age—younger patients showing higher methylation levels (6). This repetitive element demethylation may lead to increased genomic instability and transcriptional regulatory disorders, creating epigenetic conditions for keloid progression. Chromatin accessibility analysis further revealed extensive changes in chromatin open regions and transcriptomes in keloid dermal fibroblasts, closely related to gene expression changes (7). Of particular note, breast cancer susceptibility gene 1 (BRCA1) is significantly downregulated in keloid dermal fibroblasts; its knockout promotes normal dermal fibroblast proliferation and impairs migration ability, mechanistically achieved by reducing BRCA1 binding affinity and chromatin accessibility in the neuronal pentraxin 2 (NPTX2) enhancer region (7). This discovery links DNA damage repair genes to keloid pathogenesis, expanding our understanding of disease epigenetic regulatory networks.

### 3.2 N6-Methyladenosine (m6A) modification

A New Dimension of RNA Epigenetics m6A modification, as the most abundant internal modification of eukaryotic mRNA, plays a key regulatory role in keloid pathogenesis. Keloid tissue exhibits a high m6A modification state, associated with imbalanced expression of m6A methyltransferases and demethylases (8–12). Specifically, m6A demethylase FTO is elevated in keloid, promoting collagen deposition and fibrosis progression by regulating m6A modification and stability of COL1A1 mRNA (10). Conversely, m6A methyltransferase KIAA1429 is downregulated; its overexpression inhibits fibroblast migration and reduces COL1A1 and α-SMA levels, while knockdown produces opposite effects; mechanistically, KIAA1429 participates in keloid formation regulation by maintaining m6A modification and stability of TGF-β1 mRNA (13). Zinc finger CCCH domain containing protein 13 (ZC3H13) similarly accelerates keloid formation by mediating m6A modification and stability of homeodomain-interacting protein kinase 2 (HIPK2) mRNA (11). Heterogeneous nuclear ribonucleoprotein C (HNRNPC) is overexpressed in keloid tissue and fibroblasts, enhancing stability of m6A-modified WD repeat domain 77 (WDR77) mRNA by recognizing m6A modifications, thereby upregulating TGF-β and SMAD3 expression to promote fibroblast migration and proliferation (9). ALKB homolog 5 (ALKBH5) promotes keloid formation by reducing m6A-YTHDF2mediated reticulocalbin 1 (RCN1) mRNA degradation, activating IRE1α-XBP1mediated endoplasmic reticulum stress (8). These studies collectively depict the complex regulatory network of m6A modification in keloid, providing multiple potential targets for developing RNA epigenetic-targeted therapeutic strategies.

Aberrant epigenetic remodeling described above does not independently drive keloid fibrosis; instead, it serves a critical upstream regulatory function to tune the transcriptional intensity and duration of all core fibrotic signaling cascades detailed in the following section, bridging epigenetic alterations to functional cellular phenotypes.

This epigenetic-signaling cross-talk eliminates the isolated division between epigenetic regulation and intracellular signal transduction, and lays a molecular foundation for subsequent immune, metabolic and mechanical signal integration.

## 4 Core signaling pathways: molecular engines of fibrotic cascade reactions

### 4.1 TGF-β/Smad pathway: the master switch of fibrosis regulation

The transforming growth factor-β (TGF-β)/Smad signaling pathway is the most classical and central regulatory axis in keloid pathogenesis. This pathway is overactivated in keloid tissue and fibroblasts, driving excessive extracellular matrix (ECM) deposition and tissue fibrosis (13–17). TGF-β1 activates downstream Smad2/3 phosphorylation by binding to TGF-β receptor II (TβRII), forming Smad2/3/4 complexes that translocate to the nucleus to regulate target gene transcription (15). Ubiquitin-specific peptidase 11 (USP11) amplifies the TGF-β/Smad signaling cascade and enhances proliferation, invasion, migration, and collagen deposition capabilities of keloid-derived fibroblasts by deubiquitinating TβRII to enhance its stability; USP11 knockout significantly inhibits keloid growth with minimal impact on normal skin fibroblasts, suggesting its specific advantage as a therapeutic target (15). Tumor necrosis factor-α stimulated gene-6 (TSG-6) inhibits keloid fibroblast proliferation and induces apoptosis by interfering with TGF-β1/Smad signal transduction, involving mechanisms that reduce Smad2/3 phosphorylation levels, block Smad2/3/4 complex formation and nuclear translocation, and upregulate inhibitory Smad7 expression (16). Activating transcription factor 3 (ATF3) is significantly elevated in keloid tissue, enhancing fibroblast proliferation, invasion, and collagen synthesis capabilities while inhibiting apoptosis by promoting TGF-β1 and basic fibroblast growth factor 2/8 (FGF2/8) production (17). These findings reveal the complexity of TGF-β/Smad pathway regulation and provide theoretical basis for multi-target interventions.

### 4.2 MAPK/ERK pathway: the driver of proliferation and migration

The mitogen-activated protein kinase (MAPK)/extracellular signal-regulated kinase (ERK) pathway is another key signaling axis regulating keloid fibroblast proliferation, migration, and ECM synthesis. This pathway is continuously activated in keloid, forming cross-talk with the TGF-β/Smad pathway to jointly drive pathological processes (18–20). Silent information regulator 6 (SIRT6) overexpression significantly inhibits keloid fibroblast proliferation, invasion, and collagen synthesis while promoting apoptosis, mechanisms closely related to inhibiting MAPK/ERK pathway activity; epidermal growth factor (EGF, MAPK/ERK pathway agonist) treatment can reverse SIRT6’s inhibitory effects, confirming this pathway’s central position in the SIRT6 regulatory network (18). Protein tyrosine phosphatase 1B (PTP1B) is reduced in keloid tissue and fibroblasts; its overexpression inhibits cell proliferation, motility, ECM synthesis, and MAPK/ERK pathway activity, while knockdown produces opposite effects. ERK inhibitors can block PTP1B knockdown-induced phenotypic changes, proving that PTP1B maintains fibroblast homeostasis through negative regulation of the MAPK/ERK pathway (19). Serum deprivation protein response (SDPR) is significantly downregulated in keloid fibroblasts; its overexpression blocks TGF-β1/Smad pathway activation through ERK1/2-dependent mechanisms, inhibiting cell proliferation, motility, and ECM production, providing new perspectives for understanding cross-regulation of signaling pathways in keloid (20).

### 4.3 PI3K/AKT/mTOR pathway: the center of cell survival and metabolic regulation

The phosphatidylinositol 3-kinase (PI3K)/protein kinase B (AKT)/mammalian target of rapamycin (mTOR) pathway integrates extracellular signals to regulate cell survival, proliferation, metabolism, and protein synthesis, playing multiple roles in keloid pathogenesis (21–25). Sunitinib, as a multi-target tyrosine kinase inhibitor, induces cell cycle arrest, significant apoptosis, and ECM component expression inhibition in keloid fibroblasts through complete inhibition of the PI3K/AKT/mTOR pathway, including complete inhibition of type I and III collagen and upregulation of autophagy-related protein LC3B; in keloid xenograft nude mouse models, sunitinib induced complete graft regression, with efficacy significantly superior to commonly used intralesional injection drug triamcinolone acetonide (21). cAMP directly activated exchange protein (Epac1) regulates keloid fibroblast function through the AKT signaling pathway; Epac inhibitor ESI-09 reduces cell proliferation and migration capabilities in a concentration-dependent manner, induces apoptosis, and significantly reduces AKT phosphorylation levels (22). USP37 promotes keloid fibroblast growth, invasion, migration, ECM accumulation, and glycolysis by deubiquitinating and stabilizing Spalt-like transcription factor 4 (SALL4), thereby activating the PI3K/AKT pathway; PI3K pathway inhibitor LY294002 can block SALL4-mediated pro-fibrotic effects (23). DJ-1 protein activates the PI3K/AKT/mTOR pathway by undergoing transnitrosylation reactions with phosphatase and tensin homolog (PTEN) through Snitrosylation modification, reducing PTEN phosphatase activity, thereby promoting keloid cell proliferation, migration, and invasion; after DJ-1 Cys106 site mutation blocks S-nitrosylation, these effects are eliminated, suggesting that targeting DJ-1 Snitrosylation may be a potential therapeutic strategy (24). Vestigial-like family member 3 (VGLL3) is highly expressed in keloid fibroblasts, promoting glycolysis and collagen production through activation of the Wnt/β-catenin signaling pathway, while WNT2 knockdown can reverse VGLL3’s pro-fibrotic effects (25).

### 4.4 Wnt/β-catenin and Hippo/YAP pathways: integration points of mechanical force-biochemical signals

Wnt/β-catenin and Hippo/Yes-associated protein (YAP)/transcriptional coactivator with PDZ-binding motif (TAZ) pathways are key nodes integrating mechanical force signals with cellular biochemical reactions, playing unique roles in keloid pathogenesis (12, 26–31). Syndecan 1 (SDC1) promotes keloid fibroblast migration and invasion through Wnt/β-catenin signaling pathway-mediated epithelial mesenchymal transition (EMT); SDC1 knockdown significantly inhibits cell migration and invasion capabilities, reduces N-cadherin and vimentin expression, and upregulates E-cadherin expression, suggesting SDC1’s potential as a therapeutic target (26). Methyl-CpG binding protein 2 (MeCP2) activates the Wnt/β-catenin pathway by binding to the ADAM12 promoter to regulate its expression, promoting keloid fibroblast proliferation, migration, invasion, and ECM accumulation (27). WNT5A drives IL-6-dependent EMT processes through the JAK/STAT pathway to promote keloid pathogenesis; WNT5A-activated fibroblasts or keloid fibroblast-secreted IL-6 activate adjacent keratinocyte JAK/STAT signaling, inducing EMT marker expression; WNT5A silencing or IL-6 neutralizing antibody blockade can reverse STAT pathway overactivation and EMT phenotypes (28). circPTPN12 activates the Wnt signaling pathway by sponging miR-21-5p, promoting keloid fibroblast growth; circPTPN12 knockdown inhibits cell proliferation, migration, and invasion, induces apoptosis, while miR-21-5p inhibitors can partially reverse these effects (29). YAP/TAZ show high expression and high nuclear localization in keloid fibroblasts; targeted knockdown of YAP or TAZ significantly inhibits cell proliferation, reduces migration, induces apoptosis, and downregulates COL1A1 production; YAP/TAZ inhibitor verteporfin shows more potent inhibitory effects, suggesting therapeutic potential for targeting the Hippo pathway (30). Periostin (POSTN), as one of the most significantly upregulated ECM proteins in keloid, regulates fibroblast migration and collagen I/III expression through the Wnt/β-catenin pathway; siRNA knockdown of POSTN significantly inhibits keloid fibroblast migration and collagen production, confirming its value as a therapeutic target (32, 33). Keloid fibroblasts exhibit high m6A modification states, with the Wnt/β-catenin pathway being the main activated signaling cascade, and immunofluorescence staining shows this pathway is mainly activated in dermal fibroblasts (12).

The intracellular signaling cascades elaborated above do not function independently of tissue immune status; instead, they dynamically interact with immune cells and inflammatory mediators to build a self-amplifying fibrotic loop, bridging intracellular molecular transduction with tissue-level inflammatory microenvironment pathology. This regulatory circuit explains why isolated discussion of signaling pathways or immune changes cannot fully interpret keloid progression, and it also lays a foundation for integrating immune, metabolic and mechanical signals into a unified pathogenic network.

## 5 Immune microenvironment: cascade amplification from inflammation to fibrosis

### 5.1 Innate immune cells: the double-edged sword of macrophage polarization

**Figure 2:**
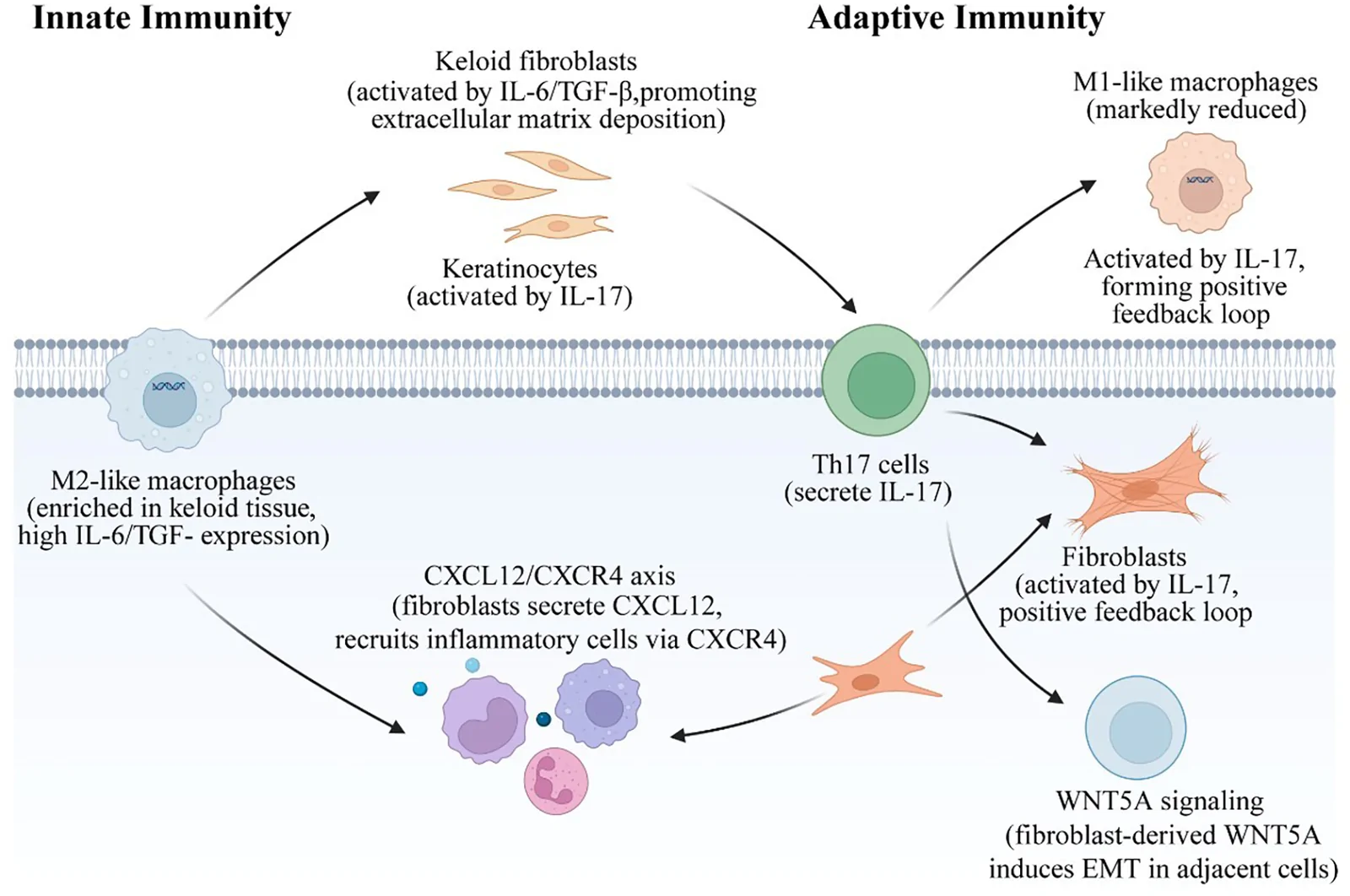
Immune microenvironment and intercellular communication in keloid: Single-cell sequencing reveals complex cellular composition in keloid tissue. Regarding innate immunity, M2-like macrophages are significantly infiltrated, promoting fibroblast activation through secretion of IL-6, TGF-β and other factors; whereas the proportion of M1 type is decreased. Regarding adaptive immunity, Th17 cells secrete IL-17, activating keratinocytes and fibroblasts to form positive feedback. In terms of cell communication, the CXCL12/CXCR4 axis mediates inflammatory cell recruitment, maintaining a chronic low-grade inflammatory state; WNT5A is secreted by fibroblasts and acts on neighboring cells to induce the EMT process. This abnormal intercellular communication is the key to maintaining the invasive growth of keloid.

The characteristic alterations of the keloid immune microenvironment are key drivers of disease occurrence and development (**Figure 2**). Single-cell RNA sequencing (scRNA-seq) analysis shows that macrophage proportions are significantly higher in keloid tissue than surrounding normal tissue, while conventional type 2 dendritic cells (cDC2) are significantly reduced; pseudotime trajectory analysis reveals two macrophage modules (Module 2 high expression of RNASE1, C1QA, CD163, CD14, C1QC, FCGRT, MS4A7; Module 10 high expression of APOC1, CTSB, CTSL, TYROBP) exhibiting tumor-associated macrophage characteristics, upregulated in latestage keloid cells (34). M1 and M2 macrophages play diametrically opposite roles in keloid pathogenesis: M2-like macrophages enhance the fibrotic phenotype of keloid fibroblasts, while M1-like macrophages exhibit anti-fibrotic effects even in pro-fibrotic environments (35, 36). ELISA detection shows reduced M1 marker CD68 expression in keloid tissue, while M2 marker CD163 is significantly elevated; for every 1 ng/mL increase in M1 levels, keloid risk decreases 0.99-fold, while for every 1 unit increase in M2 levels, risk increases 2.01-fold (36). This finding suggests that restoring M1/M2 balance to favor M1 phenotype may be an effective strategy for keloid prevention and treatment. Adipose tissue-derived stem cell (ASC)-secreted insulin-like growth factor binding protein-7 (IGFBP-7) exerts anti-keloid effects by inhibiting the BRAF/MEK/ERK signaling pathway; IGFBP-7 expression in keloid tissue is significantly lower than normal skin, and recombinant IGFBP-7 treatment or ASC coculture inhibits keloid fibroblast proliferation, promotes apoptosis, reduces angiogenesis, and downregulates pro-fibrotic factor expression including TGF-β1, vascular endothelial growth factor (VEGF), collagen I, IL-6, and IL-8 (37).

### 5.2 Adaptive immune cells: abnormal activation of T cell subsets

T cell-mediated adaptive immune responses play important roles in keloid pathogenesis. Keloid lesions and non-lesional skin show unique transcriptomic characteristics compared to healthy controls, with T cell-related immune markers significantly upregulated (38). In keloid tissue, T cell markers CD2, CD3D, CD3E, T cell/NK cell activation/migration markers CCL19, CCR7, GZMA, GZMB, GZMK, Th1 markers OASL, MX1, CCL4, Th2 markers IL4R, OX40/TNFRSF4, OX40L/TNFSF4, and Th17/22 markers S100A7, S100A8, S100A9, CCL20 are all significantly elevated (38). Notably, keloid patient skin tissues show higher expression levels of costimulatory molecules CD28, CD80, CD86, CD40L than healthy populations, with significantly increased CD8+ T cell numbers; CD8+ T cell co-culture with keloid-associated fibroblasts significantly reduces the latter’s number and viability, suggesting that CD8+ T cells may drive keloid-associated immune suppression (39). The roles of T helper cell 17 (Th17) and IL-17 in keloid pathogenesis are receiving increasing attention: systematic reviews show that all 13 studies meeting inclusion criteria confirm increased Th17 cell infiltration and elevated IL-17 expression, with IL17 amplifying inflammation and fibrotic reactions by promoting IL-6 expression, upregulating fibrotic markers through SDF-1 and HIF-1α pathways, and activating the TGF-β/Smad pathway in keloid fibroblasts (40). IL-17 induces autophagy dysfunction in keloid fibroblasts, promoting inflammatory cell death and fibrosis through STAT3 and HIF-1α-dependent signaling pathways; defects in autophagosome-autolysosome transformation are associated with increased necroptosis and fibrosis progression, and HIF-1α inhibitors can reverse these pathological changes (41).

### 5.3 Inflammatory factor networks: maintenance of chronic low-grade inflammation

Keloid tissue exhibits a chronic low-grade inflammatory state, with multiple inflammatory factors forming complex regulatory networks. Chemokine CXCL12 is elevated in keloid scars, associated with reduced dipeptidyl peptidase-4 (DPP4) expression; keloid-derived fibroblast autocrine TGF-β1 negatively regulates DPP4 expression, inhibiting DPP4’s reduction of extracellular CXCL12 levels, leading to CXCL12 accumulation in keloid fibroblasts (42). Most circulating leukocytes and peripheral blood and tissue-infiltrating inflammatory cells express C-X-C motif chemokine receptor 4 (CXCR4) rather than CXCR7, suggesting that CXCL12-driven chemotaxis is mainly mediated through CXCR4 (42). The TGF-β/DPP4/CXCL12 axis recruits inflammatory cells through CXCR4 receptors, promoting keloid chronic inflammation. Interleukin family members play multiple roles in the keloid inflammatory microenvironment: IL-6, IL-8, IL-10, IL-11, IL-13, IL-17, etc., are all abnormally expressed in keloid tissue, participating in regulating fibroblast activation, ECM synthesis, and immune cell recruitment (40, 41, 43, 44). The role of glutamate metabolism in keloid inflammatory reactions has recently been revealed: transcriptome analysis shows significant enrichment of neuroactive ligand-receptor interaction pathways in keloid tissue, with glutamate receptor subunit GRIN2D significantly upregulated and mainly expressed in fibroblasts; metabolomics detection shows significantly elevated glutamate and glutamine levels in keloid tissue; glutamate stimulation of fibroblasts significantly enhances expression and secretion of inflammatory cytokines IL-6 and IL-11, as well as chemokines CXCL2, CXCL3, and CXCL8 (IL-8) (44). These findings reveal the potential role of the neuro-immunefibrosis axis in keloid pathogenesis.

Single-cell sequencing reveals clear spatial immune heterogeneity in keloids: profibrotic M2 macrophages concentrate in lesion cores, while anti-fibrotic M1 macrophages mostly reside at lesion edges. Spatial transcriptomics verifies localized paracrine cross-talk between immune cells and fibroblasts via IL-17 and CXCL12, forming a regional pro-fibrotic microenvironment. However, published spatial profiling data for human keloids remain scarce; integrated single-cell and spatial multi-omics will be key to locating immune-fibroblast interactive niches for targeted therapy.

The sustained inflammatory signals generated by the disordered immune microenvironment do not act independently of intracellular metabolic homeostasis; instead, inflammatory mediators secreted by immune cells reshape the energy and lipid metabolism of keloid fibroblasts, while abnormal metabolic products in turn sustain and aggravate chronic inflammation, creating a mutually reinforcing pathological cycle linking immunity and metabolism. This reciprocal regulatory loop eliminates the isolated description of immune and metabolic abnormalities, and further connects the immune-metabolic axis to mechanical transduction signals to form an integrated multilayered pathogenic network of keloid.

## 6 Metabolic reprogramming: Warburg effect and mitochondrial dysfunction

### 6.1 Aerobic glycolysis: energy characteristics of keloid cells

**Figure 3:**
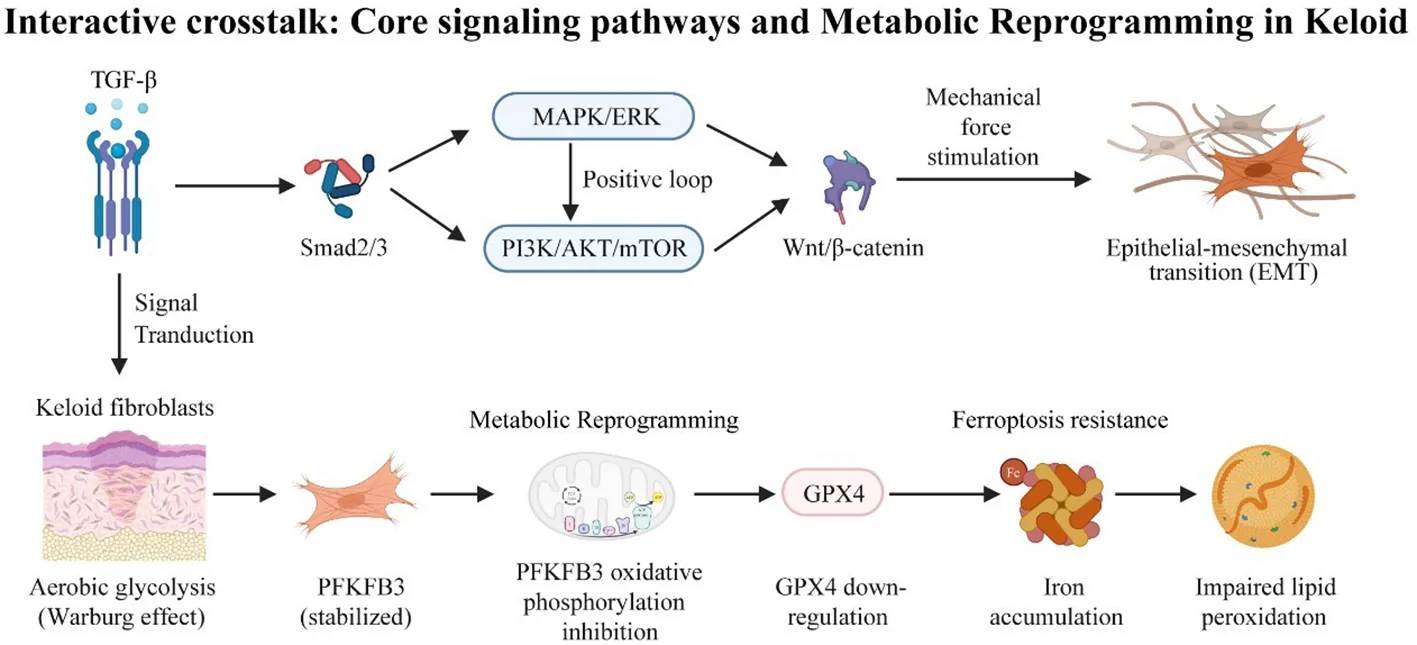
Cross-talk between core signaling pathways and metabolic reprogramming in keloid: Signal Transduction Axis: TGF-β1 binds to receptors to activate Smad2/3, forming positive feedback loops with the MAPK/ERK and PI3K/AKT/mTOR pathways to jointly promote fibrosis. The Wnt/β-catenin pathway is activated under mechanical force stimulation, inducing epithelialmesenchymal transition (EMT). Characteristics of Metabolic Reprogramming: Keloid fibroblasts exhibit significant aerobic glycolysis (Warburg effect), with stable expression of the key enzyme PFKFB3 and suppression of oxidative phosphorylation. Meanwhile, cells display ferroptosis resistance characteristics, manifested as GPX4 downregulation, iron accumulation, and impaired lipid peroxidation. Interaction Mechanisms: Metabolites (such as lactate and acetyl-CoA) can serve as cofactors to regulate histone modifications and signaling pathway activity (such as HIF-1α stabilization), while signaling pathways (such as PI3K/AKT) directly regulate the expression of glycolytic enzymes and iron metabolism genes, forming a vicious cycle.

Keloid fibroblasts exhibit metabolic reprogramming characteristics similar to tumor cells, namely the “Warburg effect”—prioritizing aerobic glycolysis over oxidative phosphorylation for energy supply even under sufficient oxygen conditions (25, 45–48) (**Figure 3**). Keloid and folliculitis keloidalis nuchae (FKN) fibroblasts show significant metabolic switching: basal glycolysis and maximum glycolytic capacity are significantly increased in perilesional keloid and FKN fibroblasts (P<0.05), while mitochondrial function parameters show enhanced oxidative phosphorylation, suggesting intact mitochondrial function but glycolytic priority (45). This metabolic phenotype switching is closely related to enhanced cell proliferation and migration capabilities, and inhibiting glycolytic flux may become a potential mechanism basis for future treatment (45). Secreted protein acidic and rich in cysteine (SPARC) promotes keloid fibroblast glycolysis by activating p38γ signaling to stabilize 6-phosphofructo2-kinase/fructose-2,6-bisphosphatase 3 (PFKFB3) protein; SPARC, p38γ, and PFKFB3 are all highly expressed in keloid patients and bleomycin-induced fibrotic mice, with SPARC overexpression promoting cell proliferation, migration, collagen production, and glycolysis, while p38γ or PFKFB3 inhibition alleviating skin fibrosis (47). Long non-coding RNA (lncRNA) HOXA11-AS promotes keloid fibroblast glycolysis, proliferation, migration, invasion, and ECM accumulation by sponging miR-205-5p to upregulate forkhead box M1 (FOXM1) expression; HOXA11-AS knockdown or miR205-5p enrichment inhibits glycolytic stress, glucose consumption, and lactate production, while FOXM1 overexpression reverses these effects (49). Legumain (LGMN), identified as a lipid metabolism-related gene, is a key biomarker of keloid, overexpressed in keloid with high diagnostic performance; gene set variation analysis (GSVA) suggests LGMN may promote fibroblast proliferation and inhibit apoptosis, significantly positively correlated with M2 macrophage infiltration (46).

### 6.2 Ferroptosis and iron metabolism: regulation of novel cell death modes

Ferroptosis, as an iron-dependent, lipid peroxidation-driven programmed cell death mode, plays a dual regulatory role in keloid pathogenesis (50–55). Iron content and lipid peroxidation (LPO) levels are significantly elevated in keloid tissue and fibroblasts, with ferroptosis-related genes solute carrier family 7 member 11 (SLC7A11), glutathione peroxidase 4 (GPX4), and nuclear factor E2-related factor 2 (Nrf2) downregulated, while transferrin receptor (TFRC) is upregulated; mitochondria show ferroptosis-related pathological changes (50). Ferroptosis inhibitor Ferrostatin-1 (Fer-1) treatment reduces iron content, inhibits ferroptosis and mitochondrial dysfunction, and reduces α-SMA, collagen I and III protein expression; while ferroptosis activator Erastin produces opposite effects, confirming the existence of ferroptosis in keloid and its regulatory role in ECM deposition and fibrosis (50). lncRNA JPX regulates keloid fibroblast ferroptosis through the FUS/SLC7A11 signaling pathway: JPX is upregulated in keloid tissue and fibroblasts, promoting SLC7A11 stability and expression through FUS; JPX silencing inhibits cell viability, migration, invasion, and ECM production, but promotes ferroptosis, while Fer-1 or SLC7A11 overexpression can reverse these effects (52). 5-Aminolevulinic acid photodynamic therapy (ALA-PDT) exerts anti-keloid effects by inducing ferroptosis: clinical studies show that 4 ALA-PDT treatments (14-day intervals) reduce keloid lesion volume by 45%, with histopathology showing collagen structural remodeling; ALA-PDT directly induces keloid fibroblast ferroptosis, activating ferroptosis signaling pathways and regulating key molecules (GPX4, ACSL4, NOX-1, SLC7A11, NCOA4), upregulating ferroptosis-related factors (MMP-9, MMP-1, CCL-2), and promoting reactive oxygen species (ROS) production (53). EP300 inhibits ferroptosis and stimulates keloid fibroblast proliferation, migration, and fibrosis through the YY1/GPX4 axis; YY1 binds to GPX4 promoter sequences to regulate its expression, with YY1-induced ferroptosis dependent on the GPX4 pathway, while EP300 overexpression reverses YY1 knockdown’s inhibitory effects (56). Notably, melanin in keloid promotes fibroblast fibrosis by inducing iron overload and ferroptosis resistance: single-cell sequencing shows enrichment of pigmentation-related pathways in keloid melanocytes, with elevated melanin levels positively correlated with keloid area and severity index; melanin promotes its transfer to the dermis by increasing basal cell permeability and inflammation, thereby activating fibroblasts through iron overload and ferroptosis resistance; iron overload and ferroptosis resistance are verified in keloid patient primary fibroblasts and skin tissue, and inhibiting these processes effectively attenuates melanin-induced fibrosis (54). A new mechanism of adipocytes in keloid pathogenesis has recently been revealed: anti-ferroptotic dermal adipocytes drive keloid pathology through GPX4-mediated adipocyte-mesenchymal transition (AMT) and iron-cysteine metabolic communication; these adipocytes exhibit iron overload, ROS suppression, and impaired interferon response, with high GPX4 expression driving AMT through iron-dependent activation of TGF-β signaling pathways; GPX4activated adipocytes promote fibroblast collagen production through paracrine signals, while establishing metabolic symbiosis—adipocytes export iron to adjacent fibroblasts through solute carrier family 40 member 1 (SLC40A1), which reciprocally supply cysteine through cystathionine β-synthase (CBS)/lysosomal cystine transporter (CTNS) to maintain GPX4 activity; ferroptosis inducer RSL3 or iron chelator deferoxamine (DFO) effectively disrupts this pathological network, inhibiting GPX4/AMT and restoring interferon response, reducing keloid growth (55).

### 6.3 Sphingolipid metabolism and lipid signaling molecules

The role of sphingolipid metabolism pathways in keloid pathogenesis has recently been systematically elucidated (57–60). Analysis based on three expression profile datasets shows that 42 upregulated and 77 downregulated common differentially expressed genes in keloid fibroblasts compared to normal fibroblasts participate in ECM structural components, collagen-containing ECM, and sphingolipid metabolism pathways; 15 metabolism-related differentially expressed genes were screened, including serine palmitoyltransferase long chain base subunit 3 (SPTLC3), UDPglucose ceramide glucosyltransferase (UGCG), and sphingomyelin synthase 2 (SGMS2), all enriched in sphingolipid pathways (57). Gene set variation analysis (GSVA) shows lower glycosphingolipid (GSL) biosynthesis in keloid fibroblasts than normal fibroblasts, with qPCR confirming SPTLC3, UGCG, and SGMS2 regulation in keloid fibroblasts (57). circ_0000129 promotes keloid fibroblast proliferation and migration by sponging miR-485-3p to upregulate SGMS2 expression; circ_0000129 and SGMS2 are amplified in keloid tissue and fibroblasts, while miR-485-3p is downregulated; circ_0000129 silencing inhibits cell proliferation and migration, with miR-485-3p inhibitors reversing these effects (58). Comprehensive single-cell sequencing and machine learning analysis systematically studied the role of glycosphingolipid metabolism pathways in keloid pathogenesis for the first time: key genes including ARSA, GBA2, SUMF2, GLTP, GALC, and HEXB were screened, and constructed diagnostic models and immune infiltration analysis show this gene set can serve as new therapeutic targets for keloid; unsupervised clustering identified two keloid subgroups, with significant differences in immune cells, inflammatory factor differences, and main enriched pathways between different subgroups; single-cell resolution transcriptome landscape focused on fibroblasts, with pseudotime trajectory analysis showing increased GSL metabolism pathway activity associated with fibroblast differentiation, and cell communication network analysis revealing fibroblast-centered communication regulatory networks in keloid, with GSL metabolism pathway activity affecting intercellular communication (60). Lipids and their derivatives play multiple roles in keloid formation: arachidonic acid (AA) synthesis and non-essential fatty acid synthesis abnormalities are important mechanisms of keloid formation; butyric acid (BA), prostaglandin E2 (PGE2), prostaglandin D2 (PGD2), and other lipid derivatives form complex interactions with excessive fibrosis regulation; docosahexaenoic acid (DHA) combined with BA and 15deoxy-Δ12,14-prostaglandin J2 shows significant cytotoxicity; among sphingolipids, ceramide (Cer) has limited pro-apoptotic effects in keloid fibroblasts, while sphingosine-1-phosphate (S1P) promotes excessive fibrosis, with its analog FTY720 showing opposite beneficial effects; vitamin D and hexadecylphosphocholine (HePC) show potential anti-fibrotic and anti-proliferative properties (59).

The multiple metabolic abnormalities described above do not operate independently of tissue mechanical microenvironment; there exists a tight bidirectional regulatory circuit between metabolic programs and mechanical signal transduction, which bridges intracellular metabolic remodeling and extracellular physical tension signals and addresses the deficiency of isolated narrative for each pathological dimension. This regulatory link unifies metabolic and mechanical pathogenic modules, and integrates with the previously elaborated genetic, epigenetic and immune networks to build a complete multi-layer pathogenic framework of keloid.

## 7 Mechanical force transduction: from physical stimulation to biochemical reactions

### 7.1 Mechanical force sensitivity and matrix stiffness perception

Keloid clinically occurs at high-tension sites (chest, shoulders, earlobes, etc.), with mechanical force participating in disease occurrence as an initial factor, while matrix mechanical properties further drive disease progression, forming a vicious cycle of continuous invasion into surrounding normal tissue (61–66). Keloid fibroblasts exhibit enhanced mechanical force sensitivity and dysfunctional mechanical force transduction signaling: compared to normal fibroblasts, keloid fibroblasts have higher baseline nuclear YAP levels and are insensitive to matrix stiffness and TGF-β stimulation; TGF-β1 can induce p-SMAD3 in both cell types, proving this pathway is functionally intact and not overactivated in keloid fibroblasts; myosin inhibitor Blebbistatin inhibits α-SMA expression and cell stiffness, linking elevated YAP signaling to the keloid phenotype (67).

Beyond the canonical Hippo-YAP/TAZ transcriptional relay axis, the mechanosensitive ion channel PIEZO1 acts as the primary upstream mechanical sensor that initiates stretch-triggered calcium influx, a key molecular component omitted in prior mechanistic summaries. PIEZO1 is a plasma membrane cation channel specifically activated by membrane tension and cyclic stretch; upon mechanical loading, channel conformation opens rapidly to permit robust extracellular Ca²_+_ entry, serving as the core upstream trigger of the abnormal calcium signaling observed in keloid fibroblasts. PIEZO1 is significantly overexpressed in fibroblasts isolated from hightension keloid predilection sites, and pharmacological blockade of PIEZO1 fully abolishes the exaggerated calcium spike responses under cyclic stretching, confirming its indispensable role in keloid mechanotransduction. Functionally, PIEZO1-mediated calcium overload acts upstream of YAP/TAZ nuclear translocation: sustained high intracellular Ca²_+_ stabilizes YAP protein and impedes its cytoplasmic retention, creating a synergistic PIEZO1-Ca²_+_-YAP/TAZ mechanocascade that amplifies fibrotic gene transcription (COL1A1, α-SMA) under persistent skin tension.

Cyclic stretching induces abnormal calcium influx in keloid-derived fibroblasts: stretching microdevice experiments show that the keloid group has a significantly higher positive response rate to mechanical stretching than the control group (15.7% vs 8.2%, P = 0.029), with significantly higher calcium concentration peaks in responsive cells (2.20 vs 1.26, P = 0.0022), cutoff value 1.77, and 10.4% of keloid-derived fibroblasts showing high calcium spikes beyond normal ranges (64). Long-term cyclic stretching experiments further confirm that keloid group calcium spike amplitudes are significantly higher than the control group (1.66 vs 1.41, P = 0.02), cutoff value 2.12, with 9.6% of keloid-derived fibroblasts showing multiple high calcium spikes (65). These findings suggest that abnormal calcium signaling may be a marker of keloid specific subpopulations, and normalizing excessive intracellular calcium concentrations may become a therapeutic target. Facial and neck keloid distribution is related to skin thickness and stiffness changes: clinical analysis of 113 patients shows keloid most commonly occurs at the mandibular angle (41.3%) and lateral neck (20.0%) regions, with these high-incidence areas not significantly associated with sebaceous gland dense areas and acne-prone areas; binomial logistic regression shows posture related skin hardness and thickness changes significantly predict keloid distribution, with skin hardness and thickness changes associated with prolonged mechanical force (posture changes) most significant at high keloid predilection sites (62).

### 7.2 Mechanical force-biochemical signal conversion mechanisms

Mechanical force signals are converted to biochemical reactions through multiple molecular mechanisms, driving keloid pathological processes. Alpha-cardiac muscle actin 1 (ACTC1), as a cytoskeletal target, participates in keloid mechanical force transduction: bioinformatics analysis and *in vitro* experiments show ACTC1 is significantly upregulated in keloid tissue and fibroblasts; siACTC1 effectively reduces keloid fibroblast proliferation, invasion capabilities, and F-actin, α-SMA, and collagen I levels; lipoic acid-modified konjac glucomannan (KGM-LA)-based adhesive hydrogel loaded with siACTC1 significantly inhibits keloid growth in mouse models through sustained ACTC1 inhibition and reduced mechanical tension (68). Mechanical force-induced increases in TGF-β and type I collagen suggest the TGF-β/Smad pathway may be activated through mechanical stimulation; proteomics analysis-screened key molecules related to cell signaling and oxidative stress significantly change after cyclic mechanical stimulation; keloid fibroblast cytoskeletal network components increase after mechanical stimulation (63).

Metabolic activity studies of keloid nodules reveal the energetic basis for their continuous expansion: analysis of 57 nodules shows higher vascular density at nodular edge areas than central areas, with fibroblast density significantly higher than surrounding connective tissue (190.29 ± 64.45 vs 140.18 ± 63.94, P<0.05); individual nodule area increases at 2–4 years, then decreases to approximate horizontal lines at 17 years, but total nodule number/dermal area ratio increases with disease duration, with maximum ratio of 48.01% in the 17-year group; nodules grow continuously within keloid lesions through increasing number and total area rather than individual nodule volume increase, playing important roles in energy metabolism activities (48).

The PIEZO1-Ca ² _+_-YAP/TAZ mechanotransduction cascade described above cannot function independently of post-transcriptional RNA regulatory programs mediated by lncRNAs, circRNAs and miRNAs. Mechanical tension and intracellular calcium signals dynamically remodel the expression profiles of diverse non-coding RNAs, while non-coding RNAs in turn tune the abundance and activity of core mechanosensory and fibrotic molecules, forming a bidirectional regulatory loop that bridges physical mechanical stimuli with intracellular RNA epigenetic control. This mutual regulatory relationship eliminates the isolated description of mechanical signaling and RNA regulatory modules, and integrates non-coding RNAs into the unified genetic-epigenetic-immune-metabolic-mechanical multidimensional pathogenic network of keloid.

## 8 Non-coding RNAs: a new dimension of fine regulatory networks

### 8.1 Long non-coding RNAs

**Figure 4:**
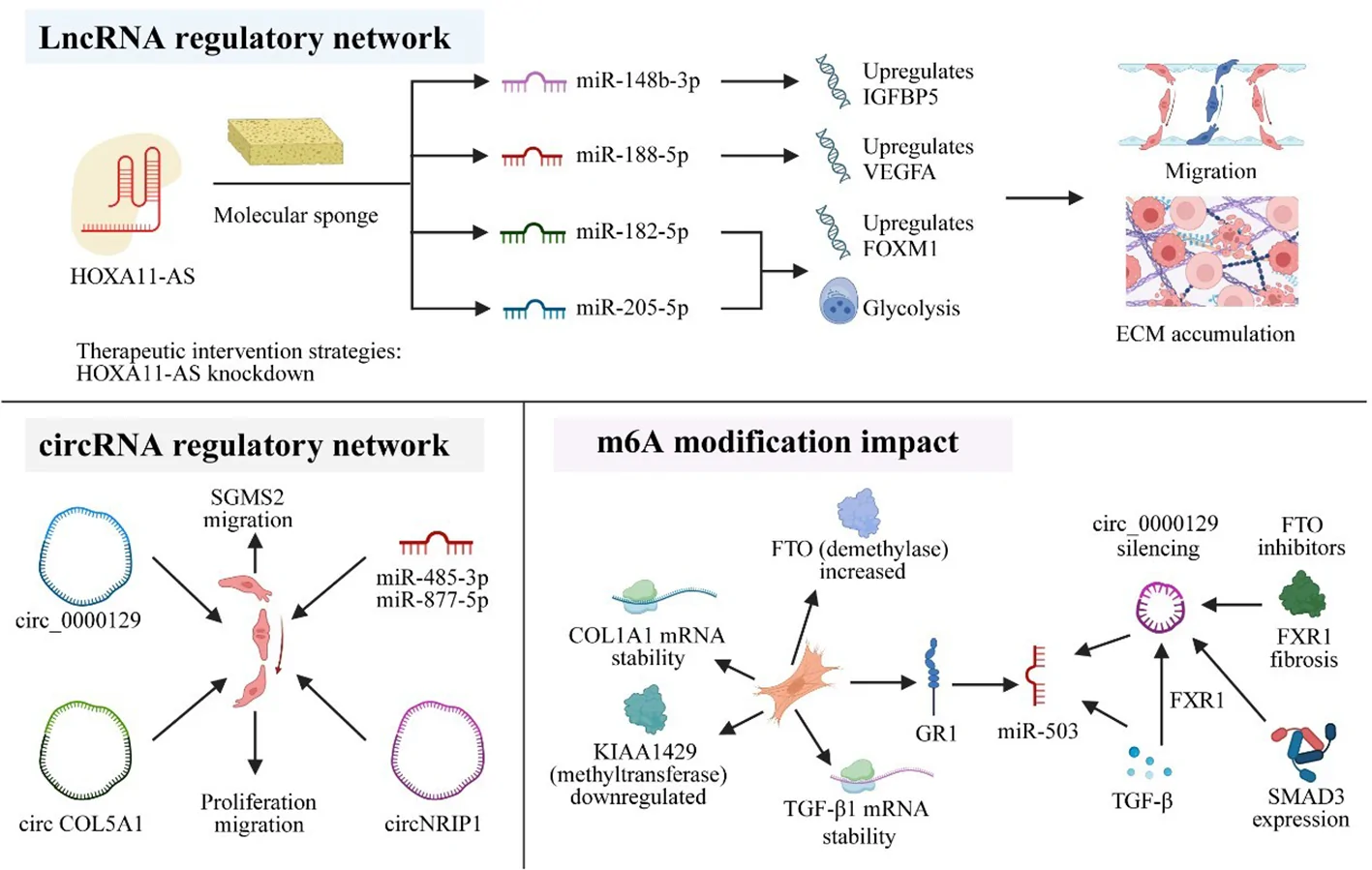
Regulatory networks of non-coding RNAs in keloid: This figure illustrates the main regulatory networks of lncRNAs, circRNAs, and miRNAs in keloid. lncRNA Regulatory Network: HOXA11-AS serves as a “molecular sponge” to adsorb multiple miRNAs, including miR-148b-3p (upregulating IGFBP5), miR-182-5p (upregulating ZNF217), miR-188-5p (upregulating VEGFA), and miR-205-5p. (upregulating FOXM1), thereby promoting glycolysis, proliferation, migration, and ECM accumulation. circRNA Regulatory Network: circ_0000129 sponges miR-4853p to upregulate SGMS2, promoting proliferation and migration; circCOL5A1 sponges miR-877-5p to upregulate EGR1, promoting ECM production; circNRIP1 promotes fibrosis through FXR1-mediated maturation of miR-503. Effects of m6A Modification on RNA Regulation: FTO (demethylase) is elevated, promoting COL1A1 mRNA stability; KIAA1429 (methyltransferase) is downregulated, reducing TGF-β1 mRNA stability; HNRNPC enhances WDR77 mRNA stability by recognizing m6A-modified WDR77 mRNA, promoting TGF-β and SMAD3 expression. Therapeutic intervention strategies are annotated in the figure: such as HOXA11-AS knockdown, circ_0000129 silencing, FTO inhibitors, etc., providing a molecular basis for non-coding RNAtargeted therapy.

lncRNAs play diverse regulatory roles in keloid pathogenesis, influencing fibroblast biological behaviors through multiple molecular mechanisms (49, 69–80) (**Figure 4**). Nuclear enriched abundant transcript 1 (NEAT1) promotes keloid fibroblast progression through the miR-196b-5p/FGF2 axis: NEAT1 increases in keloid tissue and fibroblasts, while miR-196b-5p decreases; NEAT1 knockdown or miR-196b-5p overexpression inhibits cell viability, migration, and ECM component production, with miR-196b-5p being a NEAT1 target and FGF2 a direct target of miR-196b-5p (70).

HOXA11-AS promotes keloid formation through multiple miRNA axes: upregulating insulin-like growth factor binding protein 5 (IGFBP5) expression by sponging miR148b-3p, promoting fibroblast proliferation, migration, and inhibiting apoptosis (71); upregulating zinc finger protein 217 (ZNF217) by sponging miR-182-5p, exerting similar pro-fibrotic effects (73); upregulating vascular endothelial growth factor A (VEGFA) by sponging miR-188-5p, promoting cell proliferation, cell cycle progression, and migration (81); upregulating forkhead box M1 (FOXM1) by sponging miR-2055p, promoting glycolysis, proliferation, migration, invasion, and ECM accumulation (49). YY1-induced HOXA11-AS activates oxidative stress and inflammation through epigenetic modification of the Nrf2 pathway: HOXA11-AS is upregulated in keloid, with silencing reducing fibroblast proliferation, migration, fibrosis, and oxidative stress; HOXA11-AS promotes Nrf2 promoter methylation through EZH2-mediated DNMT1 recruitment, with YY1 enhancing HOXA11-AS transcription by binding to its promoter (82). lncRNA H19 promotes keloid formation through the miR-769-5p/eukaryotic translation initiation factor 3A (EIF3A) pathway: H19 expression increases in keloid tissue and fibroblasts, with knockdown hindering cell proliferation, migration, invasion, ECM deposition, and enhancing apoptosis (74). lncRNA ZNF252P-AS1/miR-15b-5p promotes fibroblast proliferation by regulating the BTF3-STAT3 signaling pathway: BTF3 is upregulated in keloid tissue, miR-15b-5p overexpression inhibits BTF3 and downstream molecule expression, inhibiting cell proliferation and migration while upregulating E-cadherin; high ZNF252P-AS1 expression inhibits miR-15b-5p, activating the JAK2/STAT3 pathway (76). lncRNA B3GALT5-AS1 inhibits keloid progression through β-Trcp1-mediated HuR ubiquitination: B3GALT5-AS1 is significantly downregulated in keloid tissue and fibroblasts, with overexpression inhibiting cell proliferation, glycolysis, invasion, and migration, promoting apoptosis; B3GALT5-AS1 binds HuR and reduces its stability through β-Trcp1-mediated ubiquitination (75). lncRNA CPEB2-AS1 is identified as a novel molecular marker for keloid diagnosis, highly expressed in keloid tissue, with functional experiments showing it significantly affects cell proliferation, migration, invasion, and apoptosis (78). lncRNA RNF144A-AS1 promotes keloid fibrosis through P300-mediated PDE4 promoter H4K5 acetylation: RNF144A-AS1 binds HuR to promote P300 expression, with P300 upregulating histone H4K5 acetylation of the PDE4 promoter to enhance PDE4 expression (77). NEDD4L inhibits keloid fibroblast proliferation and migration by regulating YY1 ubiquitination-mediated glycolytic metabolic reprogramming: NEDD4L is downregulated in keloid, with overexpression inhibiting cell viability, proliferation, and migration, promoting YY1 degradation; YY1 induces glycolysis through transcriptional regulation of hexokinase 2 (HK2) (80).

### 8.2 Circular RNAs

CircRNAs regulating keloid pathogenesis as miRNA sponges have been a research hotspot in recent years (37, 49, 83–91). hsa_circ_0038382 upregulates T-box transcription factor 5 (TBX5) to inhibit keloid formation by sponging miR-940: this circRNA and TBX5 are reduced in keloid, while miR-940 is elevated; circRNA overexpression inhibits fibroblast proliferation, migration, and invasion, while TBX5 knockdown reverses these effects (83). circCOL5A1 promotes keloid fibroblast proliferation, migration, invasion, and ECM production through the miR-877-5p/early growth response 1 (EGR1) axis: circCOL5A1 is upregulated in keloid tissue and fibroblasts, with silencing inhibiting cell function and promoting apoptosis (84). circFoxp1 promotes cell proliferation, migration, and ECM deposition by binding to RACK1: this circRNA is highly expressed in keloid tissue, localized in the cytoplasm, with overexpression enhancing cell function and inhibiting apoptosis, and RNA sequencing showing it is related to cell proliferation, inflammatory response, and oxidative phosphorylation (86). The circ_0000129/miR-485-3p/SGMS2 axis regulates keloid fibroblast proliferation and migration: circ_0000129 and SGMS2 are amplified in keloid, while miR-485-3p is downregulated; circ_0000129 silencing inhibits cell function, with miR-485-3p loss reversing these effects (58). circ_0057452 promotes keloid progression by sponging miR-7-5p to upregulate GRB2-associated binding protein 1 (GAB1): circ_0057452 and GAB1 are upregulated in keloid, while miR-7-5p is downregulated; circRNA knockdown inhibits cell function, with miR-7-5p inhibitors reversing these effects (90). circNRIP1 promotes keloid progression through FXR1mediated upregulation of miR-503-3p and miR-503-5p: circNRIP1 is highly expressed in keloid tissue, with knockdown blocking cell proliferation and ECM-related protein expression, increasing apoptosis rates; mechanistically, circNRIP1 maintains FXR1 stability by blocking Fbxo4-mediated fragile X messenger ribonucleoprotein protein 1 (FXR1) ubiquitination and degradation, with FXR1 promoting pre-miR-503 maturation (89). circSLC8A1 inhibits keloid fibroblast growth, migration, and ECM deposition through the miR-181a-5p/HIF1AN pathway: this circRNA is downregulated in keloid, with overexpression inhibiting cell function and promoting apoptosis (91). hsa_circ_0002198 is significantly elevated in keloid tissue and fibroblasts, with EIF4A3 binding to its flanking regions to promote circRNA production and its regulatory role in fibroblast progression (87). circRNA expression profiling analysis during wound healing in keloid-prone individuals identified 29 differentially expressed circRNAs, constructing 14 ceRNA networks, with circ_064002 regulating fibronectin1 (FN1) expression by competing with miR-30a/b-5p, and circ_064002 knockdown downregulating FN1 expression and inhibiting cell viability, migration, invasion, and repair capabilities (85). High-throughput sequencing identified differentially expressed circRNAs, miRNAs, and mRNAs in 37 keloid and 37 normal skin tissue samples, with an average of 120 circRNAs upregulated, 12 downregulated, 44 miRNAs upregulated, 63 downregulated, 656 mRNAs upregulated, and 156 downregulated; hsa_circ_0001320 and circCOL5A1 upregulation were verified, and hsa_circ_0001320 interaction with miR-574-5p was confirmed (88).

### 8.3 microRNAs

miRNAs finely regulate keloid pathogenesis-related gene expression through posttranscriptional regulation (92–95). miR-29a-3p and its target genes (COL1A1, COL1A2, COL3A1, COL5A1, COL5A2) are differentially expressed in keloid, with miR-29a-3p overexpression reducing target gene expression and knockdown increasing their expression; these genes perform well in keloid diagnosis, especially when using keloid non-lesional skin or normal scar tissue as controls (92). miR-26a-5p mediates salidroside’s anti-keloid effects by inhibiting JAG1: JAG1 is upregulated in keloid tissue, with salidroside treatment inhibiting its expression; salidroside depletes keloid fibroblast viability, inhibits proliferation and migration, but stimulates apoptosis, with JAG1 knockdown enhancing these functional effects; miR-26a-5p interacts with JAG1 3’UTR, and its inhibition abolishes JAG1 knockdown effects (93). miR-23b-3p is upregulated in keloid tissue, primary fibroblasts, and KEL FIB cell lines, with miR23b-3p inhibition promoting apoptosis and inhibiting proliferation and collagen I, III, and fibronectin expression; tumor necrosis factor alpha-induced protein 3 (TNFAIP3, A20) is a direct target of miR-23b-3p, and its inhibition abolishes miR-23b-3p inhibitor effects; miR-23b-3p inhibitors reduce receptor-interacting serine/threonine-protein kinase 1 (RIPK1) expression, partially reversed by A20 inhibition (94). miR-194-5p exerts inhibitory effects in keloid fibroblasts by targeting nuclear receptor subfamily 2 group F member 2 (NR2F2): miR-194-5p is reduced in keloid fibroblasts, with overexpression inhibiting cell invasive phenotype, and NR2F2 knockdown and miR194-5p overexpression producing similar effects; NR2F2 knockdown effectively reverses miR-194-5p inhibitor-mediated effects (95).

The widespread ceRNA regulatory networks formed by lncRNAs, circRNAs and miRNAs do not operate independently of cell fate control; non-coding RNAs serve as master upstream modulators of multiple programmed cell death programs, while apoptosis and ferroptosis signaling in turn reshape the transcription and stability of noncoding RNAs, establishing a closed regulatory loop linking RNA fine-tuning and cell survival/death. This regulatory relationship unifies non-coding RNA regulation and cell death mechanisms into a complete pathological module, and together with the genetic, immune, metabolic and mechanical axes elaborated previously, constructs the multilayer integrated network of keloid pathogenesis.

## 9 Cell death modes: from apoptosis to novel programmed death

### 9.1 Apoptosis resistance and cell survival advantage

One of the most significant biological characteristics of keloid fibroblasts is resistance to apoptotic stimuli, a feature that confers tumor-like persistent growth capabilities (16, 17, 96). Secreted clusterin (sCLU) plays a key role in keloid pathogenesis: bioinformatics analysis identified 3 keloid-related apoptosis genes (ANLN, CLU, SFRP2) from 122 differentially expressed genes, with CLU consistently reduced in both training and validation datasets; experimental validation shows significantly reduced sCLU in keloid fibroblasts, accompanied by BAX downregulation and BCL2 upregulation; sCLU overexpression significantly promotes apoptosis, reduces cell viability, upregulates BAX, and downregulates BCL2 and fibrotic factor genes (collagen I, III, α-SMA, CTGF), suggesting sCLU’s potential as a tissue-level biomarker and therapeutic target (96). ATF3 promotes keloid fibroblast growth and invasion and inhibits apoptosis through the TGF-β/Smad pathway: ATF3 upregulates BCL2 levels, inhibits BCL2-associated death promoter (Bad), Caspase3, and Caspase9 expression, enhancing cell invasive potential (170). TSG-6 induces keloid fibroblast apoptosis and inhibits proliferation through the TGF-β1/Smad pathway: TSG-6 blocks Smad2/3/4 complex formation and nuclear translocation, upregulates Smad7 expression, and inhibits plasminogen activator inhibitor-1 (PAI-1) (16).

### 9.2 Ferroptosis: iron-dependent lipid peroxidation-driven cell death

The role of ferroptosis in keloid pathogenesis has been detailed in previous sections, with its potential as a novel therapeutic target becoming increasingly prominent (50–56). Based on bioinformatics analysis, 10 cuproptosis-related genes (CRGs) were identified for keloid diagnosis, with GSVA analysis showing high CRG expression affects ECM receptor interaction-related gene sets (97). In-depth analysis of ferroptosis-related molecular mechanisms provides a theoretical basis for developing therapeutic strategies to induce keloid cell ferroptosis.

In summary, the pathogenesis of keloid involves multiple dimensions, including genetic susceptibility, epigenetic regulation, aberrant signaling pathways, immune microenvironment disorders, metabolic reprogramming, dysregulated mechanical force transduction, and fine-tuning by non-coding RNAs. **Table 1** systematically summarizes these key molecular biomarkers, providing a reference for understanding the molecular basis of the disease and developing targeted therapies. Based on the in-depth understanding of these molecular mechanisms, therapeutic strategies for keloid have been shifting from single-modality surgery/hormone injection toward multi-target combination interventions in recent years. The following sections will systematically elaborate on emerging therapeutic strategies based on pathogenic mechanisms.

**Table 1:** Key molecular biomarkers in keloid pathogenesis.

Category	Molecule name	Expression of change	Function/mechanism	References
genetic susceptibility	HLA-DRB1*15	Risk allele	Associated with an increased risk of keloid scarring, involving immune regulation	(1)
genetic susceptibility	MUC4	High-frequency mutation (55.6%)	Enrichment in cancerrelated microRNA and calcium signaling pathways	(2)
Epigenetic regulation	FTO	raise	m6A demethylase promotes COL1A1 mRNA stability and collagen deposition	(10)
Epigenetic regulation	ALKBH5	raise	By reducing the degradation of RCN1 mRNA mediated by m6AYTHDF2, the endoplasmic reticulum stress mediated by IRE1α- XBP1 is activated	(8)
Core signaling pathway	TGF-β1/Smad	activate	By translocating the Smad2/3/4 complex into the nucleus, it regulates the transcription of target genes and drives excessive deposition of ECM	(13–17)
Core signaling pathway	YAP/TAZ	Increased nuclear localization	Enhanced mechanosensitivity, converting mechanical force into biochemical signals through the Hippo pathway	(30)
immune microenvironment	CD163 (M2)	raise	The marker of M2-like macrophages is associated with a 2.01-fold increased risk of keloid formation	(36)
immune microenvironment	IL-17	raise	Promoting inflammatory cell death and fibrosis through STAT3 and HIF1α-dependent signaling pathways	(40, 41)
metabolic reprogramming	PFKFB3	raise	The key enzyme of glycolysis, regulated by SPARC/p38γ, promotes	(47)
metabolic regulation	GPX4	Downregulation (of ferroptosis resistance)	Key inhibitory factors of ferroptosis, downregulation of which leads to ferroptosis resistance and ECM deposition	(50, 56)
non-coding RNA	HOXA11-AS	raise	As a multi-miRNA sponge, it promotes glycolysis, proliferation, and ECM accumulation through multiple miRNA axes	(49, 70, 71, 73)
non-coding RNA	circ_0000129	raise	Upregulating SGMS2 through the adsorption of miR-485-3p promotes the proliferation and migration of keloid fibroblasts	(58)

## 10 Therapeutic targets and emerging treatment strategies

### 10.1 Small molecule compounds and targeted drugs

**Figure 5:**
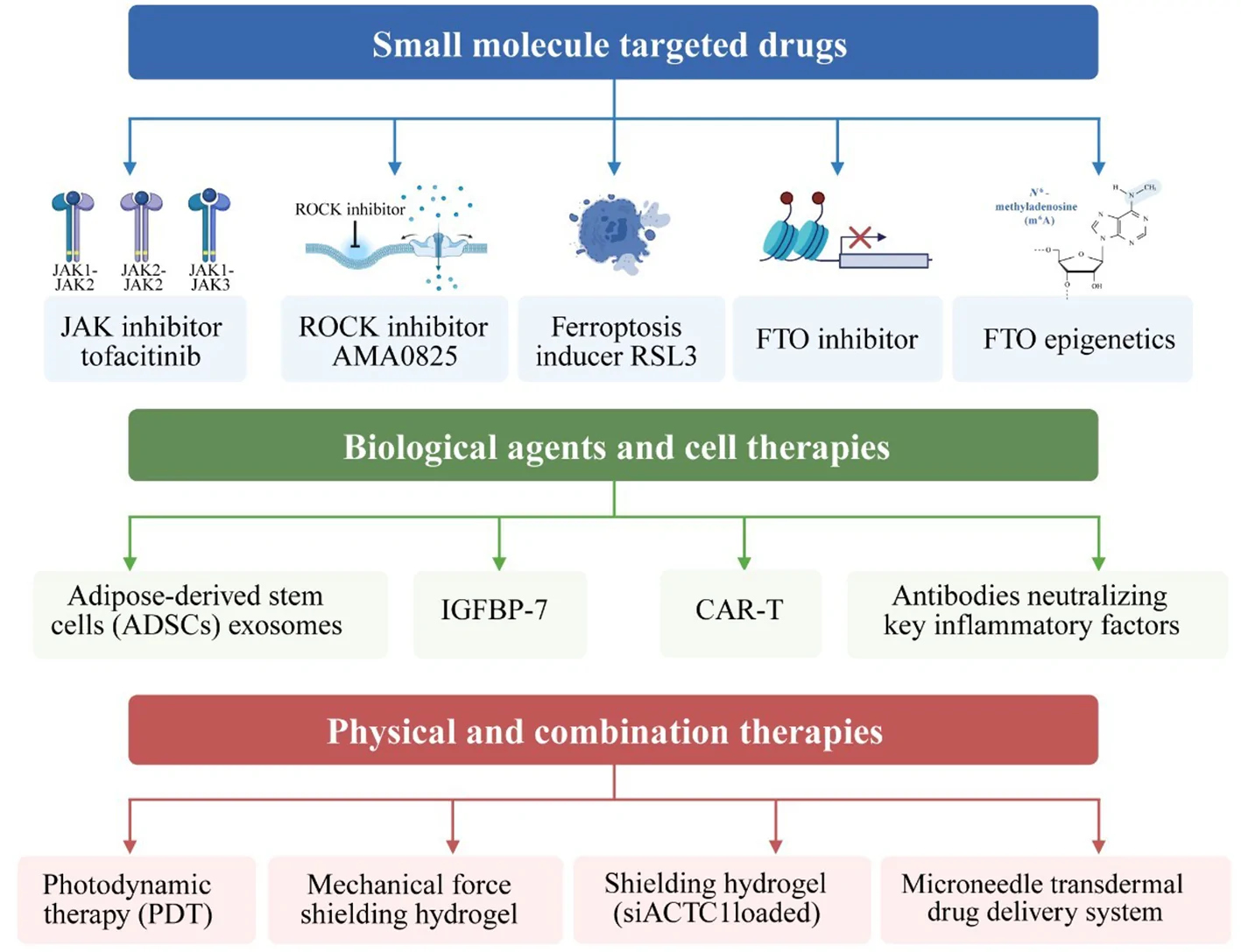
Schematic diagram of precision treatment strategies for keloid based on multidimensional mechanisms: Based on the analysis of pathogenic mechanisms, treatment strategies are shifting from single surgery/hormone injection to multi-target combination therapy. (1) Small Molecule Targeted Drugs: Interventions targeting signaling pathways (such as JAK inhibitor tofacitinib, ROCK inhibitor AMA0825), metabolism (ferroptosis inducer RSL3), and epigenetics (FTO inhibitors). (2) Biologics and Cell Therapy: Utilizing exosomes secreted by adipose-derived stem cells (ADSCs) or IGFBP-7 for immune modulation; utilizing CAR-T or antibodies to neutralize key inflammatory factors. (3) Physical and Combination Therapies: Including photodynamic therapy (PDT) to induce cell death, mechanical force-shielding hydrogel (siACTC1-loaded), and microneedle transdermal drug delivery systems. These strategies aim to break the vicious cycle of “inflammation-fibrosis-mechanical tension”.

Based on deep understanding of keloid pathogenesis, multiple small molecule compounds and targeted drugs demonstrate therapeutic potential (21, 98–111) (**Figure 5**). ROCK inhibitor AMA0825 shows excellent anti-proliferative activity *in vitro*, ex vivo, and three-dimensional spheroid models, with a half-maximal growth inhibitory concentration (IC50) of 28.19 ± 1.6 nM, significantly superior to dexamethasone (35.35 ± 2.6 μM) and triamcinolone acetonide (37.84 ± 3 μM); this drug induces cell cycle G1 phase arrest and produces synergistic effects when combined with dexamethasone (98). 2-Methoxyestradiol (2ME2) reduces keloid fibroblast proliferation levels by targeting p38 to inhibit the MAPK/Erk signaling pathway, with p38 inhibitors and 2ME2 both reducing proliferation levels, suggesting 2ME2 exerts anti-proliferative effects by targeting p38 (99). The oral JAK1 and JAK3 kinase inhibitor tofacitinib was evaluated in a single-arm clinical trial in Chinese keloid patients, showing significant improvements in Patient and Observer Scar Assessment Scale (POSAS), Vancouver Scar Scale (VSS), and Dermatology Life Quality Index (DLQI) scores after 12 weeks of treatment (p<0.05), with reduced keloid volume, lesion height, and dermal thickness, and significantly reduced collagen I, Ki67, p-STAT3, and p-SMAD2 expression; *in vitro* experiments confirmed tofacitinib inhibits fibroblast proliferation and collagen I synthesis by inhibiting STAT3 and SMAD2 pathways (100). The role of vitamin D signaling pathways in keloid has received attention: CYP24A1 (vitamin D metabolic enzyme) is significantly overexpressed in keloid keratinocytes, with ketoconazole (nonspecific cytochrome P-450 inhibitor) reducing keloid and normal keratinocyte proliferation, while specific CYP24A1 inhibitor VID400 significantly affecting only keloid keratinocyte proliferation; both inhibitors reduce pro-fibrotic gene (periostin, hyaluronan synthase 2) expression in keloid cells, with vitamin D combined with CYP24A1 inhibitors enhancing effects on target genes (101). β-sitosterol (SIT) inhibits keloid proliferation, migration, and invasion by regulating the PTEN/PI3K/AKT signaling pathway: network pharmacology and molecular docking show robust binding affinity between SIT and PTEN, with SIT treatment reducing p-PI3K and p-AKT levels, downregulating vimentin and Snail proteins, and upregulating occludin-1 and Ecadherin; YS-49 (PI3K agonist) reverses SIT’s inhibitory effects, with *in vivo* experiments showing SIT inhibits growth in nude mouse keloid models and increases PTEN expression (102). Artesunate inhibits keloid fibroblast proliferation by targeting the IRE1α/XBP1 signaling pathway and reducing TGF-β1: the IRE1α/XBP1 pathway is activated in keloid fibroblasts, with IRE1α knockdown inhibiting TGF-β1 expression, cell viability, and cell cycle progression; artesunate treatment significantly reduces cell proliferation, with cell cycle arrest in G1 phase (103). Scutellarin inhibits keloid fibroblast progression by targeting the EGFR/PI3K/AKT signaling pathway: network pharmacology analysis reveals 91 overlapping targets, with KEGG enrichment analysis showing significant association with the PI3K/AKT pathway, and molecular docking and dynamics simulations confirming strong binding affinity between scutellarin and AKT1/EGFR; *in vitro* experiments show scutellarin significantly inhibits cell proliferation, migration, and collagen production (104). Astragalin (AST) inhibits keloid fibroblast proliferation, motility, and ECM synthesis and regulates the MAPK pathway: AST treatment hinders proliferation, migration, and invasion capabilities of primary keloid and normal fibroblasts, with keloid fibroblasts showing greater sensitivity to AST, and AST inhibiting ECM deposition and inactivating MAPK signaling pathways (105). Novel curcumin derivative DMC-HA more effectively inhibits keloid fibroblasts than curcumin: CCK-8 experiments show DMC-HA more effectively inhibits proliferation at equal concentrations, with flow cytometry analysis showing it more significantly induces apoptosis; DMC-HA increases ROS production, upregulates Bax, cleaved PARP, and cleaved Caspase-3, with antioxidant NAC reversing its effects; DMC-HA also inhibits IL-6-induced p-STAT3 increase (106). Halofuginone (HF) regulates keloid fibroblast responses to TGF-β-induced fibrosis: 50 nM HF impairs cell proliferation, reduces TGF-β1-induced α-SMA and type I procollagen expression, reduces migration, prevents matrix contraction, and increases metalloprotease/inhibitor (MMP/TIMP) ratios (107). Derazantinib inhibits keloid fibroblast biological activity through FGFR signaling: this selective FGFR inhibitor inhibits cell proliferation, migration, invasion, and collagen production, significantly inhibiting plasminogen activator inhibitor-1 (PAI-1) transcription and expression closely related to collagen deposition and tissue fibrosis, and reducing implanted keloid weight (108). Salidroside blocks keloid fibroblast invasive progression by upregulating miR-26a-5p to inhibit JAG1 (93). Chlorpheniramine maleate (CPM) exerts anti-keloid activity by regulating the IL-6/JAK1/STAT3 signaling pathway: CPM inhibits cell proliferation and migration, promotes apoptosis, and blocks G0/G1 phase; intralesional injection significantly reduces scar in nude mouse keloid models, with RNA sequencing analysis showing IL-6 and JAK-STAT signaling pathways negatively correlated with control groups (109). Indocyanine green-mediated photodynamic therapy (ICG-PDT) for keloid: photodynamic mechanisms maximally inhibit cell activity and migration, induce autophagy and apoptosis, inhibit collagen synthesis, achieving therapeutic effects at low drug concentrations, with clinical cases using surgery combined with modified ICG-PDT obtaining better treatment outcomes (110). Parecoxib (COX-2 inhibitor) reduces primary keloid fibroblast growth and Bcl-2 protein levels: treatment significantly reduces cell growth, decreases viability, increases fragmented nuclei, with Bcl-2 reduced and activated Caspase-3 increased (111).

### 10.2 Biologics and cell therapy

Biologics and cell therapy have opened new avenues for keloid treatment [122128]. Single-cell transcriptomic analysis identified therapeutic subpopulations of adipose-derived mesenchymal stromal cells (ASCs) for treating human keloid: NOG and IL6 upregulation strongly correlates with keloid fibroblast formation, while APP and NOTCH1 increase correlates with keloid keratinocyte development; functional scRNA-seq analysis identified ASC subpopulations capable of inhibiting keloid fibroblast, keloid keratinocyte, or both developments, with pig ASCs effectively preventing hypertrophic scar formation in miniature pig hypertrophic scar models (112). Botulinum toxin A (BTX-A) promotes transdifferentiation of primary keloid myofibroblasts into adipocyte-like cells: BTX-A enhances adipocyte markers PPARγ and C/EBPα expression, increases lipid droplet accumulation, and reduces α-SMA, collagen I and III expression; BTX-A enhances BMP4 and p-smad1/5/8 expression, with Noggin (BMP4 antagonist) reversing BTX-A-mediated effects (113). ASCderived IGFBP-7 prevents keloid formation by inhibiting the BRAF/MEK/ERK signaling pathway: IGFBP-7 is significantly lower in keloid tissue than normal skin, stimulating keloid fibroblasts to reduce proliferation, increase apoptosis, reduce angiogenesis, and inhibit TGF-β1, VEGF, collagen I, IL-6, IL-8, BRAF, MEK, and ERK expression (37). Different effects of M1 and M2 macrophages on keloid fibroblasts: M2-like macrophages enhance the fibrotic phenotype, while M1-like macrophages have anti-fibrotic effects; restoring M1/M2 balance to favor M1 may be a therapeutic strategy (35). Exosomes and conditioned medium in keloid and hypertrophic scar treatment: mesenchymal stem cell exosomes from different sources inhibit or support keloid and hypertrophic scar progression through multiple mechanisms, with comprehensive analysis of MSC exosome effects from different sources having important value (114). Patient-derived keloid xenograft (PDKX) model simulates keloid disease: transplanting keloid patient peripheral blood mononuclear cells (PBMCs) and keloid lesions into NOD/SCID/IL-2Rγnull mice, compared to traditional models, PDKX model shows neotissue formation along transplanted skin edges, significantly elevated lymphocyte infiltration and collagen synthesis, significantly elevated human Th17 cells, IL-17, HIF-1α, and chemokine levels, and significantly increased keloid lesion weight (115). ADSCs inhibit keloid fibroblast proliferation and promote apoptosis by suppressing ITGA2: consensus gene coexpression network analysis identifies ITGA2 as a novel marker in PPI networks, with ADSC conditioned medium inhibiting keloid fibroblast proliferation and increasing apoptosis, and ITGA2 overexpression reversing ADSC-induced apoptosis increase and proliferation reduction (116).

### 10.3 Physical therapy and combination strategies

Physical therapy and combination strategies hold important positions in keloid management (53, 66, 68, 117–120). Tissue clearing and three-dimensional imaging of keloid vascular systems: using optical tissue clearing methods combined with lightsheet and confocal fluorescence microscopy to detailedly characterize keloid vascular three-dimensional structures; compared to normal skin, keloid scar papillary and upper reticular layers have higher vascular density, while lower reticular layer vessels are vertically arranged but have lower density, with excessive vascularization mainly induced in papillary and upper reticular layers, potentially promoting keloid pathogenesis (118). Drug delivery strategies controlling angiogenesis imbalance: angiogenesis is inevitable in wound healing, but uncontrolled angiogenesis may initiate keloid; anti-angiogenic factors for postoperative prevention, with various strategies including steroid injection, surgical excision, radiotherapy, pressure therapy, cryosurgery, etc., but with high recurrence rates; drug delivery strategies controlling angiogenesis may achieve keloid-free wound healing (117). 5-Fluorouracil (5-FU)loaded carboxymethyl chitosan nanoparticle microneedle transdermal delivery: CMC nanoparticles increase normal human fibroblast viability to 150%, while 5-FU-loaded CMC nanoparticles significantly inhibit keloid fibroblasts to 16%; nanoparticles inhibit TGF-β1, with dissolution and diffusion confirmed in pig dorsal skin models, and this system has self-administered painless treatment potential (119). Physics-informed neural networks (PINNs) learning keloid *in vivo* mechanical inhomogeneity based on shear wave elastography: designing ultrasound-compatible hydrogel covering test keloid, transforming free boundaries into manageable interfaces; finite element simulation and phantom experiments validate methods, with *in vivo* experiments confirming clinical practicality, successfully inferring keloid spatial shear modulus, creating diverse keloid elasticity maps, improving diagnosis, treatment, and prevention (66). Novel needle-type electrocoagulation combined with drug therapy: needle assisted electrocoagulation combined with corticosteroid and 5-FU injection effectively removes keloid, with energy consumption and duration tests showing higher energy and longer duration producing larger ablation zones and higher surface temperatures; combination therapy significantly reduces keloid volume in nude mouse models, upregulates MMP-1 and MMP-3, downregulates COL I and COL III, inhibits angiogenesis and proliferation; clinical trial of 6 patients shows significant reduction in VSS and POSAS scores, with no significant adverse events (120).

Based on the aforementioned multidimensional pathogenic mechanisms, current keloid treatment is evolving toward a precision intervention system targeting specific molecular targets and pathological processes. Each therapeutic strategy is designed against the core pathogenic axes elaborated above, yet universal translational barriers restrict clinical application: Small-molecule inhibitors: Target fibrosis/metabolic pathways with clear molecular basis, but most data come from simplified cell/animal models inconsistent with human scar microenvironment; systemic toxicity and poor intralesional drug distribution limit clinical efficacy. Cell and biological therapies: Mildly regulate immune imbalance with low toxic risk, but high production costs and inadequate long-term safety data hinder wide clinical use. Physical combined therapies: Block mechanical fibrotic signals unique to keloids, yet shallow penetration and inconsistent operation standards lead to unstable treatment outcomes. Collectively, current single-target interventions cannot fully break the self-amplifying fibrotic loop. The main gaps for clinical translation include insufficient humanized disease models, lack of stratified clinical trial evidence, and immature local drug delivery systems. Future multi-target combined regimens and optimized biomaterials are required to bridge basic mechanisms and clinical practice. **Table 2** systematically categorizes treatment strategies according to their underlying pathogenic mechanisms, encompassing small-molecule targeted drugs, biologics, metabolic interventions, physical therapies, and combination strategies, thereby providing a reference framework for clinical decision-making and translational research. The development of these therapeutic strategies relies indispensably on reliable disease models and technological platforms. The following sections will introduce *in vitro* models, three-dimensional culture systems, animal models, and technical advances in keloid research.

**Table 2:** Treatment strategies for keloid based on pathogenic mechanisms.

Target/strategy	Representative drugs/methods	Mechanism of action	Research phase	References
TGF-β/Smad pathway	Triamcinolone acetonide	Inhibit Smad2/3 phosphorylation, block the formation of Smad2/3/4 complex and nuclear translocation	clinical application	(16)
ROCK pathway	AMA0825	Inhibiting cell proliferation and inducing G1 phase arrest, it is superior to dexamethasone and triamcinolone acetonide	preclinical	(98)
JAK	tofacitinib	Inhibit the STAT3 and SMAD2 pathways to reduce the expression of collagen I, Ki67, p- STAT3, and pSMAD2	Phase II clinical trial	(100)
PI3K/AKT/mTOR pathway	Sunitinib	Fully inhibit the PI3K/AKT/mTOR pathway, induce apoptosis, and suppress the expression of ECM components	preclinical	(21)
glycolysis	2-DG	Inhibit the Warburg effect, reduce glucose consumption and lactate production	preclinical	(45)
Ferroptosis induction	RSL3, Erastin	Induce ferroptosis, reduce iron content, and inhibit lipid peroxidation	preclinical	(50, 53)
photodynamic therapy	ALA-PDT, ICG-PDT	Inducing ferroptosis and apoptosis, regulating key molecules (GPX4, ACSL4, NOX-1, SLC7A11, NCOA4)	Clinical exploration	(53, 110)
cell therapy	ADSCs, ASCs	Paracrine inhibition, IGFBP-7 inhibits fibroblast proliferation through the BRAF/MEK/ERK signaling pathway	Clinical exploration	(37, 112)
Mechanical force regulation	siACTC1 hydrogel	Reduce cell stiffness and mechanical tension by inhibiting ACTC1	preclinical	(68)
m6A modification	Targeting FTO/ALKBH5	Restore RNA modification balance and regulate the expression of target genes such as COL1A1 and TGF-β1	conceptual phase	(8, 10)

## 11 Research models and technical advances

### 11.1 *In vitro* models and three-dimensional culture systems

Establishment of keloid research models is crucial for mechanism analysis and drug screening (121–124). Establishment of keloid-derived immortalized fibroblast (KDIF) cell lines overcomes limitations of primary cell passage senescence and limited tissue supply: KDIF cell lines were established from primary keloid fibroblasts (PKF) by transfecting human telomerase reverse transcriptase (hTERT) gene, including site-specific cell lines from edge, middle, and top regions; KDIF cell lines exhibit site-specific keloid fibroblast characteristics, with significantly reduced β-galactosidase positive cells, growth curves showing significantly faster growth characteristics, top and middle KDIF cell lines showing migration trends similar to site-specific PKF, edge KDIF cell lines showing significantly enhanced migration, all KDIF cell lines expressing keloid-related fibrotic marker collagen I protein, triamcinolone functional testing inhibiting cell migration, and ATCC short tandem repeat analysis verifying them as representative keloid cell lines (121). Three-dimensional model analysis of keloid fibroblast activation protein alpha (FAP-α) expression: FAP-α, as a serine protease involved in keloid formation, shows different expression in 2D culture and 3D Matrigel models; keloid fibroblasts (KEL FIB) show higher viability than normal human dermal fibroblasts (NHDF) in all three models, with FAP-α showing different expression in normal and keloid fibroblasts, *in vitro* conditions affecting FAP-α expression in NHDF cells but not KEL FIB cells, suggesting FAP-α expression differs between *in vivo* and *in vitro* models (122). Assessment of spheroid model applicability for keloid fibrosis research: hypothesizing that keloid fibroblast (KFs) spheroids can be used to simulate fibrosis *in vitro*, evaluating apoptosis, myofibroblast differentiation, TGF-β1 response, ECM synthesis, and ECM-related gene regulation; surprisingly, KFs show significantly downregulated fibrotic characteristics in 3D culture, with spheroids potentially not being the most suitable model for studying keloid fibrosis, but serving as effective models for studying fibrotic cell inactivation (123). Development and application of 3D keloid spheroid models: based on keloid heterogeneity and the important role of endothelial cells in pathogenesis, evaluating novel 3D *in vitro* keloid spheroids prepared from keloid fibroblasts and endothelial cells; spheroids with higher proportions of endothelial cells show increased viability and proliferation capabilities, with patient-derived keloid spheroids showing heterogeneity that may reflect individual clinical conditions, optimal fibroblast to endothelial cell ratio of 4:1; patient-derived keloid spheroids show better keloid genetic alterations than 2D monolayer culture, with spheroids showing different responses and resistances to different drugs, and 3D keloid spheroids potentially providing effective *in vitro* models for studying disease pathogenesis and precision medicine treatment (124).

### 11.2 Animal models and clinical research advances

Development of patient-derived xenograft models and humanized mouse models provides platforms closer to clinical pathophysiology for keloid research (115). As previously mentioned, the PDKX model successfully simulates keloid inflammatory microenvironment and progressive growth characteristics by integrating patient immune cells and lesion tissue, providing reliable tools for treatment strategy assessment (115).

## 12 Future perspectives and precision medicine

### 12.1 Multi-omics integration and systems biology

Keloid pathogenesis research is entering the era of multi-omics integration and systems biology. Comprehensive application of genomics, transcriptomics (including single-cell RNA sequencing), proteomics, metabolomics, epigenomics, and microbiomics will construct panoramic molecular networks of keloid pathogenesis. Machine learning algorithms (including LASSO regression, support vector machine recursive feature elimination, random forest, artificial neural networks, etc.) play increasingly important roles in biomarker identification, diagnostic model construction, and treatment response prediction (4, 46, 78, 97, 125–134). Integrating multi-dimensional analysis methods including single-cell and bulk transcriptome analysis, Mendelian randomization, immune infiltration analysis, and network pharmacology will reveal previously undiscoverable disease mechanisms and therapeutic targets.

### 12.2 Precision medicine and individualized treatment

Precision medicine strategies based on molecular classification are the future direction of keloid treatment. Keloid heterogeneity requires individualized treatment plans based on patient genetic background, immune status, metabolic characteristics, and lesion location. Inhibitors targeting specific signaling pathways (such as TGFβ/Smad, PI3K/AKT/mTOR, JAK/STAT, etc.), regulatory strategies targeting specific cell types (such as M2 macrophages, activated fibroblast subpopulations), and intervention means based on metabolic characteristics (such as glycolysis dependence, ferroptosis sensitivity) will constitute the core content of future keloid precision treatment. Development of biomaterial-assisted drug delivery systems (such as hydrogels, microneedles, nanoparticles) will achieve targeted drug delivery and controlled release, improving efficacy and reducing side effects.

## Conclusion

Keloid is an intractable invasive scar with high recurrence, which cannot be interpreted by any single pathway alone. This review constructs an interconnected multidimensional pathogenic network and clarifies the bidirectional crosstalk among genetics, epigenetics, fibrotic signaling, immunity, metabolism, PIEZO1/YAP mechanotransduction and non-coding RNA regulatory networks, overcoming the fragmented discussion of previous studies. Genetic susceptibility lays the inherent risk, and epigenetic changes switch on abnormal transcription to overactivate TGF-β, MAPK, PI3K and Wnt/Hippo cascades. These pathways trigger chronic inflammation dominated by M2 macrophages and Th17 cells. Inflammatory factors remodel cellular metabolism to induce Warburg effect and ferroptosis resistance, while metabolic disorders in turn sustain inflammation. Mechanical force is sensed by PIEZO1 and transduced through YAP/TAZ to worsen metabolic abnormalities and matrix stiffening. lncRNAs, circRNAs and miRNAs serve as universal fine-tuners to coordinate all above processes and prevent fibroblast death. All these factors form a self-reinforcing fibrotic loop that fuels keloid onset and recurrence. Current targeted therapies only partially block isolated pathogenic steps and cannot eradicate keloid. Future work will adopt multi-omics and single-cell sequencing to dig out key hub targets, verify combination treatments via patient-derived 3D and animal models, and stratify patients by molecular profiles to realize personalized precision therapy for better long-term outcomes.

## Glossary

ACSL4: Acyl-CoA Synthetase Long Chain Family Member 4
ADAM12: A Disintegrin And Metalloproteinase 12
ADSCs: Adipose-Derived Stem Cells
ALA-PDT: 5-Aminolevulinic Acid Photodynamic Therapy
ALKBH5: AlkB Homolog 5
AMT: Adipocyte-Mesenchymal Transition
APC: Adenomatous Polyposis Coli
APOC1: Apolipoprotein C1
BAX: BCL2-Associated X Protein
BCL2: B-Cell Lymphoma 2
BMP4: Bone Morphogenetic Protein 4
BTF3: Basic Transcription Factor 3
BRAF: B-Raf Proto-Oncogene, Serine/Threonine Kinase
BRCA1: Breast
BTX-A: Botulinum Toxin A
Bad: BCL2 Associated Death Promoter
CACNA1C: Calcium Voltage-Gated Channel Subunit Alpha1 C
C1QA: Complement C1q A Chain
C1QC: Complement C1q C Chain
CASP3/Caspase3: Caspase 3
CASP9/Caspase9: Caspase 9
CAT: Catalase
CCL2: C-C Motif Chemokine Ligand 2
CCL4: C-C Motif Chemokine Ligand 4
CCL19: C-C Motif Chemokine Ligand 19
CCL20: C-C Motif Chemokine Ligand 20
CCR7: C-C Motif Chemokine Receptor 7
CCR5: C-C Motif Chemokine Receptor 5
CD14: Cluster of Differentiation 14
CD28: Cluster of Differentiation 28
CD3D/E: Cluster of Differentiation 3D/E
CD68: Cluster of Differentiation 68
CD163: Cluster of Differentiation 163
C/EBPα: CCAAT/Enhancer Binding Protein Alpha
CMC: Carboxymethyl Chitosan
COL1A1/2: Collagen Type I Alpha 1/2 Chain
COL3A1: Collagen Type III Alpha 1 Chain
COL5A1/2: Collagen Type V Alpha 1/2 Chain
COL5A1: Collagen Type V
COX-2: Cyclooxygenase-2
CPM: Chlorpheniramine Maleate
CRGs: Cuproptosis-Related Genes
CTGF: Connective Tissue Growth Factor
CTSB/L: Cathepsin B/L
CTNS: Cystinosin
CYP24A1: Cytochrome P450 Family 24 Subfamily
A Member 1;DFO: Deferoxamine;DLQI, Dermatology Life Quality Index
DMC-HA: Demethoxycurcumin-Hyaluronic Acid
DPP4: Dipeptidyl Peptidase-4
DJ-1: Parkinson Disease Protein 7
DNMT1: DNA Methyltransferase 1
Derazantinib: Derazantinib
E-cadherin: E-Cadherin
ECM: Extracellular Matrix
EGR1: Early Growth Response 1
EIF3A: Eukaryotic Translation Initiation Factor 3A
EIF4A3: Eukaryotic Translation Initiation Factor 4A3
EGFR: Epidermal Growth Factor Receptor
EMT: Epithelial-Mesenchymal Transition
ESI-09: ESI-09
ER: Endoplasmic Reticulum
ERK: Extracellular Signal-Regulated Kinase
EZH2: Enhancer of Zeste Homolog 2
FAP-α: Fibroblast Activation Protein Alpha
FCGRT: Neonatal Fc Receptor
FGF2/8: Fibroblast Growth Factor 2/8
Fbxo4: F-Box Protein 4
FKN: Folliculitis Keloidalis Nuchae
FTO: Fat Mass and Obesity-Associated Protein
FOX M1: Forkhead Box M1;FXR1, Fragile X Mental Retardation Protein 1
Fer-1: Ferrostatin-1
FGFR: Fibroblast Growth Factor Receptor
FTY720: FTY720
GAB1: GRB2 Associated Binding Protein 1
GBA2: Glucosylceramidase 2
GCDA: Glycocholic Acid
GFAP: Glial Fibrillary Acidic Protein
GLTP: Glycolipid Transfer Protein
GLS: Glutaminase
GRIN2D: Glutamate Receptor Ionotropic, N-Methyl-DAspartate 2D
GRB2: Growth Factor Receptor-Bound Protein 2
GPX4: Glutathione Peroxidase 4
GSLs: Glycosphingolipids
GZMA/B/K: Granzyme A/B/K
HIF-1α: Hypoxia-Inducible Factor 1 Alpha
HIF1AN: HIF1 Alpha Inhibitor
HLA: Human Leukocyte Antigen
HNRNPC: Heterogeneous Nuclear Ribonucleoprotein C
HIPK2: Homeodomain-Interacting Protein Kinase 2
HK2: Hexokinase 2
HOX: Homeobox
HePC: Hexadecylphosphocholine
HF: Halofuginone
ICG-PDT: Indocyanine Green Photodynamic Therapy
IGFBP-5/7: Insulin-Like Growth Factor Binding Protein 5/7
IL-6/8/10/11/13/17/22: Interleukin 6/8/10/11/13/17/22
IL-6R: Interleukin 6 Receptor
InDels: Insertions and Deletions
IRE1α: Inositol-Requiring Enzyme 1 Alpha
ITGA2: Integrin Alpha 2
IRS1: Insulin Receptor Substrate 1
JAK1/3: Janus Kinase 1/3
JPX: lncRNA JPX
JNK: c-Jun N-Terminal Kinase
KGM-LA: Lipoic Acid-Modified Konjac Glucomannan
KDIF: Keloid-Derived Immortalized Fibroblasts
KEL FIB: Keloid Fibroblasts
KIAA1429: KIAA1429
KFs: Keloid Fibroblasts
LASSO: Least Absolute Shrinkage and Selection Operator
LCM: Laser Capture Microdissection
LC3B: Microtubule-Associated Protein 1 Light Chain 3B
LGMN: Legumain
LINE1: Long Interspersed Nuclear Element-1
LncRNA: Long Non-Coding RNA
LPO: Lipid Peroxidation
LY294002: LY294002
MAPK: Mitogen-Activated Protein Kinase
MEK: Mitogen-Activated Protein Kinase Kinase
MeCP2: Methyl-CpG Binding Protein 2
MMP-1/3/9: Matrix Metalloproteinase 1/3/9
m6A: N6-Methyladenosine
MS4A7: Membrane Spanning 4-Domain Subfamily A Member 7
mTOR: Mammalian Target of Rapamycin
MX1: Myxovirus Resistance 1
N-cadherin: N-Cadherin
NEDD4/4L: Neural Precursor Cell Expressed Developmentally Down-Regulated 4/4L
NEAT1: Nuclear Enriched Abundant Transcript 1
Nrf2: Nuclear Factor E2-Related Factor 2
NOX-1: NADPH Oxidase 1
NCOA4: Nuclear Receptor Coactivator 4
NPTX2: Neuronal Pentraxin 2
NR2F2: Nuclear Receptor Subfamily 2 Group F Member 2
NSUN2: NOP2/Sun RNA Methyltransferase 2
OASL: 2’-5’-Oligoadenylate Synthetase-Like
OX40/TNFRSF4: Tumor Necrosis Factor Receptor Superfamily Member 4
OX40L/TNFSF4: Tumor Necrosis Factor Ligand Superfamily Member 4
p38γ: p38 Mitogen-Activated Protein Kinase Gamma
PAI-1: Plasminogen Activator Inhibitor-1
PARP: Poly(ADP-Ribose) Polymerase
PBMCs: Peripheral Blood Mononuclear Cells
PDE4: Phosphodiesterase 4
PDKX: Patient-Derived Keloid Xenograft
PFKFB3: 6-Phosphofructo-2-Kinase/Fructose-2,6-Bisphosphatase 3
P-gp: P-Glycoprotein
PI3K: Phosphatidylinositol 3-Kinase
PINNs: Physics-Informed Neural Networks
PIP3: Phosphatidylinositol 3,4,5-Trisphosphate
PKC: Protein Kinase C
PMSF: Phenylmethylsulfonyl Fluoride
POSTN/Periostin: Periostin
PTP1B: Protein Tyrosine Phosphatase 1B
PTPN12: Protein Tyrosine Phosphatase Non-Receptor Type 12
PDMS: Polydimethylsiloxane
PAX5: Paired Box 5
PPARγ: Peroxisome Proliferator-Activated Receptor Gamma
POSAS: Patient and Observer Scar Assessment Scale
PTEN: Phosphatase and Tensin Homolog
PTX3: Pentraxin 3
Parecoxib: Parecoxib
RACK1: Receptor for Activated C Kinase 1
RCN1: Reticulocalbin 1
RIPK1: Receptor-Interacting Serine/Threonine-Protein Kinase 1
ROCK: Rho-Associated Coiled-Coil Containing Protein Kinase
RSL3: Ras Selective Lethal 3
RNASE1: Ribonuclease A Family Member 1
RNF144A-AS1: lncRNA RNF144A-AS1
SALL4: Spalt-Like Transcription Factor 4
SAM: SAdenosylmethionine
SDC1: Syndecan 1
SDF-1: Stromal Cell-Derived Factor 1
siRNA: Small Interfering RNA
SIRT6: Sirtuin 6
SLC7A11: Solute Carrier Family 7 Member 11
SLC40A1: Solute Carrier Family 40 Member 1;SLC6A15, Solute Carrier Family 6 Member 15
SLC8A1: Solute Carrier Family 8 Member A1
SMAD2/3/4/7: SMAD Family Member 2/3/4/7
SND1: Staphylococcal Nuclease Domain Containing 1
SNVs: Single Nucleotide Variations
SFRP2: Secreted FrizzledRelated Protein 2
SIT: β-Sitosterol
SMase: Sphingomyelinase
SPTLC3: Serine Palmitoyltransferase Long Chain Base Subunit 3
SPARC: Secreted Protein Acidic and Cysteine-Rich
SGMS2: Sphingomyelin Synthase 2
SUMF2: Sulfatase Modifying Factor 2
SDPR: Serum Deprivation Protein Response
S1P: Sphingosine-1-Phosphate
STAT3: Signal Transducer and Activator of Transcription 3;sCLU, Secreted Clusterin
TAZ: Transcriptional Coactivator with PDZ-Binding Motif
TBX5: T-Box Transcription Factor 5
TβRII: TGF-β
TMB: Tumor Mutation Burden
TGF-β1/2/3: Transforming Growth Factor Beta 1/2/3
TIMP: Tissue Inhibitor of Metalloproteinase
TFRC: Transferrin Receptor
TGFA: Transforming Growth Factor Alpha
TH1/2/17/22: T Helper 1/2/17/22
TSG-6: Tumor Necrosis Factor AlphaStimulated Gene 6
TTLL12: Tubulin Tyrosine Ligase-Like 12
TYROBP: TYRO Protein Tyrosine Kinase Binding Protein
UNC5C: Unc-5 Netrin Receptor C
UGCG: UDP-Glucose Ceramide Glucosyltransferase
USP11/37: Ubiquitin-Specific Peptidase 11/37
2ME2: 2-Methoxyestradiol
2-DG: 2-Deoxy-D-Glucose
VDR: Vitamin D Receptor
VEGFA/VEGF: Vascular Endothelial Growth Factor A
VGLL3: VestigialLike Family Member 3
VSS: Vancouver Scar Scale
WDR77: WD Repeat Domain 77
WGCNA: Weighted Gene Co-Expression Network Analysis
Wnt: Wingless-Type MMTV Integration Site Family
YY1: Yin-Yang 1
XBP1: X-Box Binding Protein 1
YAP: Yes-Associated Protein
YS-49: YS-49
ZC3H13: Zinc Finger CCCH-Type Containing 13
ZNF217/252P-AS1: Zinc Finger Protein 217/252P-AS1
α-SMA: Alpha-Smooth Muscle Actin
β-Trcp1: Beta-Transducin Repeat Containing E3 Ubiquitin Protein Ligase 1.
